# Modeling the Soil Dissipation Kinetics of Small Molecules

**DOI:** 10.1021/acs.jcim.6c01812

**Published:** 2026-10-28

**Authors:** Vincent-Alexander Scholz, Richard Marchese Robinson, Sevil Payvandi, Steffen Hirte, Timothy J.C. O’Riordan, Johannes Kirchmair

**Affiliations:** † Department of Pharmaceutical Sciences, 27258 University of Vienna, Josef-Holaubek-Platz 2, Vienna 1090, Austria; ‡ Vienna Doctoral School of Pharmaceutical, Nutritional and Sport Sciences (PhaNuSpo), University of Vienna, Vienna 1090, Austria; § Syngenta Crop Protection, 101825 Jealott’s Hill International Research Centre, Bracknell, Berkshire RG42 6EY, United Kingdom

## Abstract

Understanding the metabolic fate of small molecules is central to risk assessment and the rational design of plant protection products, pharmaceuticals, cosmetics, and food additives. In environmental systems, dissipation is governed by kinetic processes operating across soil, water, and other compartments. For chemicals released into the environment, especially active ingredients of plant protection products, assessing soil persistence is an essential regulatory requirement. The ability to predict soil persistence, ideally in advance of commissioning experiments, is highly valuable for identifying novel active ingredients for plant protection products. Here, we introduce machine learning models that predict the full time course of small-molecule dissipation in soil. These models are explicitly constrained by mechanistic assumptions encoded as ordinary differential equations (ODEs). By design, this physics-constrained parametrization enforces physical plausibility. To the best of our knowledge, our models are the first to predict soil dissipation kinetics from molecular structures using a constrained time course framework. We found that this framework almost always outperformed a conventional QSAR approach of predicting prefitted kinetic parameters. We further investigated the incorporation of soil metadata, i.e., predefined and experimentally measured soil parameters, into the model, with mixed results. Our framework supports screening and design decisions by linking molecular properties and soil metadata to predicted environmental lifetimes and dissipation profiles.

## Graphical abstract

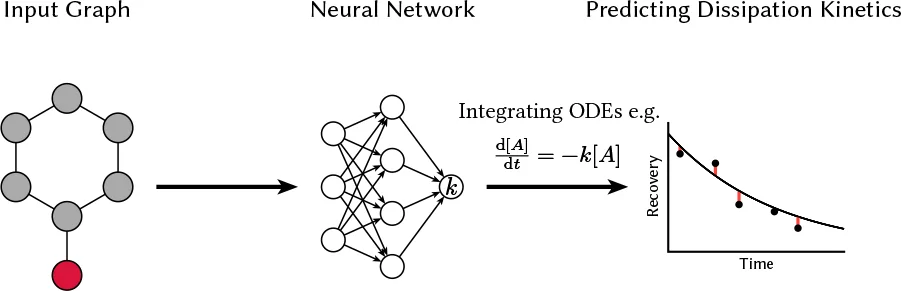

## Introduction

The duration of exposure of a biological system or environment to compounds such as plant protection products (PPPs, including pesticides and other agrochemicals), pharmaceuticals, cosmetics, and food additives is a key determinant of their efficacy and safety.^1–4^ In biological systems, the fate of a compound is governed by processes such as metabolism, distribution, and elimination, which together determine therapeutic efficacy, potential toxicity, and the overall duration of action.

In environmental systems, kinetic processes describe how compounds dissipate in soil, water, and other ecological compartments. For substances released into the environment, particularly PPP active ingredients, it is essential to understand the mechanisms driving degradation and dissipation, including microbial degradation, chemical hydrolysis, photolysis, and other abiotic processes. These processes are commonly quantified using dissipation half-lives (DT_50_; i.e., the time taken to achieve 50% dissipation) and DT_90_ values (i.e., the time taken to achieve 90% dissipation).^5^ The DT_50_ and DT_90_ values of chemicals in soil are often calculated using single first-order (SFO) kinetics, as outlined in OECD Test Guideline 307.^6^ Where study designs permit, degradation-specific half-lives (DegT_50_) may also be estimated to distinguish true degradation from broader dissipation processes.^5^

Interpreting, comparing, and working with dissipation values is nontrivial. Dissipation values can vary substantially depending on experimental conditions. For example, Latino et al.^7^ found that 31% of compounds in the Eawag-Soil data set have DT_50_ values that differ by two or more orders of magnitude, highlighting the complexity and variability of environmental metabolism. Several interacting factors can also contribute to the variable dissipation profiles for a compound.^8–12^ A major determinant is the soil microbiome: microorganisms drive compound degradation, and differences in their composition and activity can lead to markedly different degradation outcomes. Further influential parameters include soil organic matter, moisture content, soil geographic diversity, and the compound’s physicochemical properties.

Organic matter plays a dual role. On the one hand, it can reduce biodegradation by strongly adsorbing compounds, thereby limiting their bioavailability.^13^ On the other hand, it may enhance degradation by stimulating microbial growth and activity.^14^ Moisture is critical, as most biodegradation occurs in the aqueous phase. Insufficient water content can significantly slow microbial metabolism.^9^

From a modeling perspective, the dissipation kinetics of small molecules in soil are often approximated using a single-compartment model, resulting in single first-order (SFO) kinetics. This allows straightforward calculation of constant half-life (DT_50_) or DT_90_ values.^15^ However, many compounds exhibit biphasic dissipation behavior: a rapid initial phase during which the readily available fraction is lost, followed by a slow second phase characterized by the persistence of a protected fraction. This complexity necessitates more sophisticated kinetic models based on more advanced systems of ordinary differential equations (ODEs).^15^

Two commonly used kinetic models that can account for biphasic behavior are the Double-First-Order-in-Parallel (DFOP) and the Single-First-Order-Reversible-Binding (SFORB). Whereas DFOP assumes two parallel degradation pathways with distinct rate constants, SFORB accounts for the reversible binding of the compound to soil components, thereby affecting its availability for degradation. One point to note is that these kinetic models implicitly assume degradation, but are fitted to kinetic data that, to varying extents depending on the study design, may include other mechanisms of dissipation.

Whereas SFO kinetics allow a single DT_50_ or DT_90_ to be derived analytically, biphasic kinetic models (i.e., DFOP or SFORB) can yield multiple DT_50_ or DT_90_ values. For example, for the DFOP model, DT_50_ values can be derived for the fast initial phase, the slow second phase, and, via numerical methods, the overall dissipation.^15^

In the context of PPP regulation, soil dissipation kinetics is crucial for understanding biological efficacy and environmental impact. The relevance of soil persistence for environmental impact is reflected in regulation;^16^ therefore, it is crucial for researchers to design novel PPP active ingredients to anticipate challenges with soil persistence as early as possible. This includes the analysis of potential biphasic behavior. However, experimental studies are low throughput, taking several months to return results, and costly.

Hence, models capable of predicting soil dissipation from molecular structures are highly valuable to researchers seeking to discover and ensure the safety of novel PPPs. Models capable of capturing the influence of soil metadata characterizing soil conditions on dissipation would be even more valuable. Models capable of capturing biphasic behavior, rather than relying solely on SFO assumptions, would be even more valuable still. Consequently, models that accurately predict the dissipation kinetics of PPPs may lead to shorter development pipelines and reduced attrition.

Historically, two principal strategies have been employed to predict kinetics from molecular structure: (i) quantitative structure–activity relationship (QSAR) models that predict prefitted kinetic parameters, e.g., DT_50_, from molecular structure^17–24^ and (ii) time-series prediction of concentration–time profiles. To a lesser extent, machine-learning models have also been developed to predict concentration–time profiles from molecular structures by treating time as another descriptor. For example, Obrezanova et al. developed deep learning imputation models for concentration–time curves in *in vivo* pharmacokinetic data.^25^ However, they obtained mixed results when comparing the reliability of kinetic parameters derived from these predicted curves with those obtained from direct prefitting.

End point prediction via QSAR models focuses on predicting specific kinetic end points, such as soil DT_50_ for PPP active ingredients or clearance and area under the curve (AUC) for drugs. Historical data are used to compute these end points, which are then modeled using machine-learning algorithms such as random forests, support vector machines, or graph neural networks (GNNs).^17–24^ These models rely on molecular descriptors or graph-based representations to infer relationships between structure and kinetic behavior.

Time-series prediction of concentration–time profiles treats kinetics as a time-series problem, aiming to predict the full concentration–time curve from which end points can be derived. Two modeling paradigms are distinguished: unconstrained and constrained approaches.

Unconstrained approaches apply deep learning architectures such as transformers or long short-term memory (LSTM) networks directly to embedded representations of the problem, generating the time course without explicit mechanistic constraints.^25–29^ In contrast, constrained approaches integrate machine learning with systems of ordinary differential equations (ODEs).^27,30,31^ In this framework, the model predicts ODE parameters from the input encoding, and the concentration–time profile is obtained by numerically solving the equations. This enables mechanistically grounded predictions while retaining flexibility.

In this work, we compare the performance and applicability of a constrained time-series approach restricted to well-established ODEs with those of a conventional QSAR approach using early-stage dissipation data for small molecules in soil. To the best of our knowledge, this is the first application of a constrained time-course approach to predict the dissipation kinetics of small molecules in soil from molecular structure. Given empirical evidence that soil characteristics influence dissipation, we incorporate soil metadata to enhance model performance. By applying DFOP or SFORB as kinetic assumptions, a subset of the models developed can predict biphasic behavior by design. This yields expressive, informative models that remain constrained by reasonable mechanistic assumptions. Finally, we assess the models’ ability to predict soil dissipation kinetics for future studies of novel PPP active ingredients. Our work provides important insights into which modeling paradigms are most useful for predicting soil dissipation kinetics in the context of PPP active ingredient design.

## Methods

### Data Set Acquisition

#### Initial Data Set

At the beginning of this study, Syngenta shared PPP raw soil dissipation kinetics data, collected from soil metabolism studies conducted between September 2019 and May 2023, with the University of Vienna for model development and initial evaluation. The data **were** generated in accordance with a study protocol selected by Syngenta to inform the design and selection of PPP active ingredient candidates. Time courses, i.e., the percentage recovered at each time point, were generated for a variety of PPP active ingredient candidates and assay standards at time points ranging from t = 0 to 122 days. In this context, it is worth noting that the initial percentage recovery at t = 0, denoted “c0”, is measured multiple times and may be above or below 100%. While c0 should theoretically be 100%, it may not be possible to extract 100% of the applied chemical from the soil matrix, reflecting sorption associated with soil behavior. In addition, analytical issues might result in experimental artifacts, including c0 exceeding 100%. Where analytical issues arise, this is expected to give rise to high variability between the experimental replicates for c0. Values between 70 and 110% are typically considered acceptable when nonradiolabeled test items are used.^6^

The soil dissipation kinetics data originate from studies across different soils, characterized by metadata such as soil location (i.e., soil name) and soil pH measured in CaCl_2_. Full details of the types of soil metadata included in this Initial Data Set for modeling are discussed below and reported in full detail in the Supporting Information.

#### Time-Split Test Set

The University of Vienna provided Syngenta with the best-performing, optimized models for unbiased evaluation using a time-split framework, based on data from new soil metabolism studies conducted between June 2023 and August 2024, following an identical experimental protocol. Any compounds present in the Initial Data Set (e.g., assay standards) were removed from this Time-Split Test Set. None of the Time-Split Test Set data were shared with the University of Vienna, nor were the models refined after they had been shared with Syngenta based on time-split performance. These steps ensured that this Time-Split Test Set served as a completely blind validation of the models’ ability to predict future study results for novel PPP active ingredient candidates.

### Data Preprocessing: Soil Time-Courses

For model evaluation and the construction of conventional QSAR models from prefitted kinetic parameters, the experimental parameters of the kinetic ODEs were estimated using the *curve_fit* function from SciPy,^32^ enabling the derivation of end points such as DT_50_ under different kinetic assumptions (SFO, DFOP, and SFORB). Closed-form solutions exist for SFO but not for DFOP and SFORB. For DFOP and SFORB, we determined DT_50_ and DT_90_ values using a two-step approach: first, a linear search identified the upper bounds at which degradation exceeded 50% and 90% of the initial concentration. Once these bounds were established, a binary search was used to converge on the exact time points.

### Data Preprocessing: Molecular Structures and Soil Metadata Used as Input Variables

Stereochemistry-invariant InChIs (International Chemical Identifiers) were computed from the SMILES stored in the Syngenta data by specifying the SNon flag^33^ using the RDKit.^34^ These InChIs were used to ensure that the Time-Split Test Set contained no compounds or stereoisomers in common with the Initial Data Set. Where molecules could not be parsed correctly by using these steps, they were removed from the data set.

In all models investigated in this study, molecules are represented as graphs, with atoms as nodes and bonds as edges. Node features include atom types and hybridization states. When soil metadata were incorporated, these environmental descriptors were encoded as additional nodes.

A variety of soil-related metadata was examined to improve the models. In general, the metadata included pH (CaCl_2_), soil organic matter (OM), cation exchange capacity, sand content, silt content, clay content, moisture, and soil textural class. To identify the most informative features, we evaluated multiple combinations of metadata features. A complete list of metadata feature sets explored in this work is provided in the Supporting Information. To improve the numerical stability of the models, continuous features such as OM, pH, cation exchange capacity, and moisture were standardized to have a mean of 0 and a standard deviation of 1, based on their distributions in the Training Set.

### Data Splitting of the Initial Data Set for Model Development and Evaluation

After data preprocessing, the initial data set comprised 1930 time courses associated with 884 compounds. To ensure robust model evaluation, the data set was randomly split into a training set (684 compounds), a validation set (100 compounds), and a test set (100 compounds). To avoid bias introduced by assay standards, these standards were manually assigned to the training set. Furthermore, to maintain consistency across subsets and prevent leakage of compound information into the validation and test sets, all time courses corresponding to a given compound, even if from different soils, were assigned to the same split. This resulted in 1596 time courses in the training set, 153 in the validation set, and 181 in the test set.

While sufficient data to generate robust performance statistics were available to us, our use of a single train/test split did not allow us to assess the statistical significance of differences in performance metrics across the modeling approaches we performed. In contrast, Ash et al.^35^ recently proposed that multiple iterations of cross-validation be performed to assess the statistical significance of differences in performance statistics between modeling algorithms. However, this was not practical in the current context due to the computational expense of repeatedly building models across the investigated paradigms, including hyperparameter optimization (see below). Moreover, a standard cross-validation scheme would not assess differences in performance between the modeling paradigms when applied to the real-world scenario of predicting the outcomes of future studies for previously untested compounds. The time-split test set was designed to assess the latter point.

### Time-Split Test Set Details

Following data preprocessing, the time-split test set comprised 255 time courses for 210 compounds.

### Modeling Paradigms with and without Soil Metadata Features

Two GNN deep learning modeling paradigms were evaluated: (i) a constrained time-course approach, which uses a physics-based machine learning framework in which the kinetic ODEs are integrated within one of the hidden layers of the deep learning neural network and the dissipation time-courses are modeled directly; (ii) a conventional QSAR approach, in which prefitted kinetic parameters derived from the dissipation time-courses are modeled, and predicted time-courses are generated as a postprocessing step.

For both modeling paradigms, molecular graph representations were learned using graph convolution layers. As previously described, because we also built models that combine a molecular graph representation with a paradigm-specific, optimized set of soil metadata, our “conventional QSAR approach” does not strictly correspond to a pure QSAR approach when soil metadata features are incorporated.

When generating predictions, the t = 0 percentage recovery (“c0”) must be provided. For the Training Set, Validation Set, and Test Set, c0 was computed as the average of the experimental replicates. For the Time-Split Data Set, in some result sets, as indicated in the text, c0 was set to the ideal 100% to evaluate the model’s ability to predict time courses before running experiments with new compounds. However, it should be noted that all performance statistics other than those related to the raw percentage recovery values, i.e., those related to predicted DT_50_ and DT_90_ values, would be independent of the value of c0.

For models incorporating soil metadata features, experimental values were used unless otherwise noted. However, for many of these parameters, the precise values would not be known in advance of running the experiment. Hence, for the Time-Split Data Set, we also explored replacing the experimentally observed values with values based on the Training Set mode (categorical variables, e.g., soil classification) or median (numerical variables, e.g., soil pH) for each soil name, i.e., for each value of the “soil” metadatum. Coupled with setting c0 to 100%, this allowed us to examine how well these metadata-augmented models could predict soil time course and DT_50_ values before running experiments on new compounds.

For both the constrained time-course approach and conventional QSAR paradigms, modeling was repeated using three different kinetic ODE frameworks: SFO, SFORB, and DFOP.

#### Kinetic ODE Frameworks

SFO kinetics is the simplest approach to describe soil dissipation. The core assumption is that the entire amount of the compound resides in a single, homogeneous compartment, where it is fully available for dissipation via a single first-order rate constant, assumed to refer to a degradation process.^15^ The concentration over time follows the differential equation:

1 $$\frac{d\left[A\right]}{dt}=-k\left[A\right]$$

with the analytical solution:

2 $$\left[A\right]={[A]}_{0}e^{-kt}$$

where [*A*]_0_ is the initial concentration and *k* is the first-order rate constant. From this, it follows that there is a constant half-life, equivalent to DT_50_ in the current context, with an analytical solution: $\frac{\ln\;2}{k}$ . Similarly, DT_90_ has a constant, analytical solution: $\frac{\ln\;10}{k}$ (see ref 15.).

In contrast, the DFOP and SFORB frameworks^15^ account for biphasic behavior, reflecting dynamics involving more than one compartment or phase. Multiple mechanisms can give rise to biphasic dissipation. In field settings, seasonal fluctuations in temperature and moisture modulate degradation rates over time; for example, lower winter temperatures typically reduce the rate constants. The fraction available for degradation may also evolve as slow sorption and diffusion^36^ processes redistribute compounds between bioavailable and sequestered pools. Under laboratory conditions, microbial activity can decline due to limited nutrient and carbon availability, reducing the level of biodegradation. Finally, spatial heterogeneity in soils, including variation in texture, organic matter, and microbial community structure, can produce aggregate dynamics that deviate from (SFO) kinetics.^36,37^

Conceptually, DFOP models the compound as distributed between two independent compartments, each degrading according to SFO kinetics with distinct rate constants. The concentration over time follows the differential equation:

This allows for the differentiation between a fast-degrading and a slow-grading fraction. The concentration over time follows the differential equation:

3 $$\frac{d\left[A_{1}\right]}{dt}=-k_{1}\left[A_{1}\right]$$

4 $$\frac{d\left[A_{2}\right]}{dt}=-k_{2}\left[A_{2}\right]$$

with the analytical solution:

5 $$\left[A\right]={[A]}_{0}ge^{-k_{1}t}+{[A]}_{0}\left(1-g\right)e^{-k_{2}t}$$

where *g* is the fraction of the compound in the fast-degrading compartment.

SFORB assumes that, at the start of an experiment, the compound is present only in an “unprotected” compartment. From there, it may either degrade according to first-order kinetics or be reversibly transported into a “protected” compartment. One way to interpret this formalism is that some molecules become adsorbed onto soil particles.^15^ Because this adsorption can be reversible, the compound may return to the unprotected compartment. The system is described by

6 $$\frac{d\left[A_{1}\right]}{dt}=-k_{1}\left[A_{1}\right]-k_{12}\left[A_{1}\right]+k_{21}\left[A_{2}\right]$$

7 $$\frac{d\left[A_{2}\right]}{dt}=k_{12}\left[A_{1}\right]-k_{21}\left[A_{2}\right]$$

where *A*_1_ is the unbound compartment, *A*_2_ the bound (protected) compartment, and *k*_12_ and *k*_21_ are the forward and reverse binding rates. Because the analytical solution of the SFORB model is more complex than the corresponding solutions of SFO and DFOP, we include these equations in the Supporting Information.

All three frameworks (i.e., SFO, SFORB, and DFOP) were implemented as PyTorch^38^ modules by using their analytical solutions. However, it is also possible to use differential equations without analytical solutions. In such cases, a numerical solver is required.

All three frameworks are illustrated in Figure 1.

**Figure 1:**
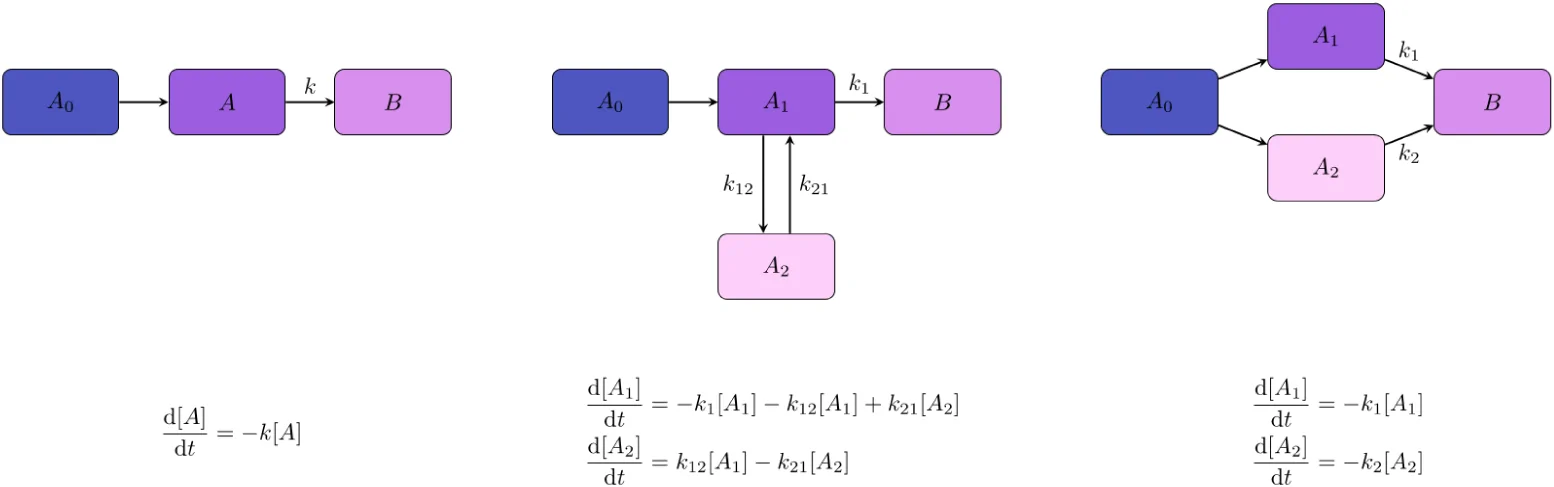
Comparison of SFO (LHS), SFORB (middle), and DFOP (RHS) kinetics, visualized as compartments.

#### Constrained Time-Course Approach

In the constrained time-series modeling approach, otherwise referred to as the “constrained time-course” or simply “time-course” approach elsewhere in the manuscript—for brevity, the analytical solutions of the ODEs (SFO, SFORB, or DFOP) were incorporated into the deep-learning architecture, and the predicted parameters (i.e., rate constants and the fraction of the compound in the fast-degrading compartment for DFOP) were used to predict the experimentally observed percentage recovery at points after t = 0. The model was optimized during training to minimize the loss computed from the differences between these values and the experimental recovery values, which are expressed as percent recovery of the applied concentration at t = 0. This framework is illustrated in Figure 2A.

**Figure 2:**
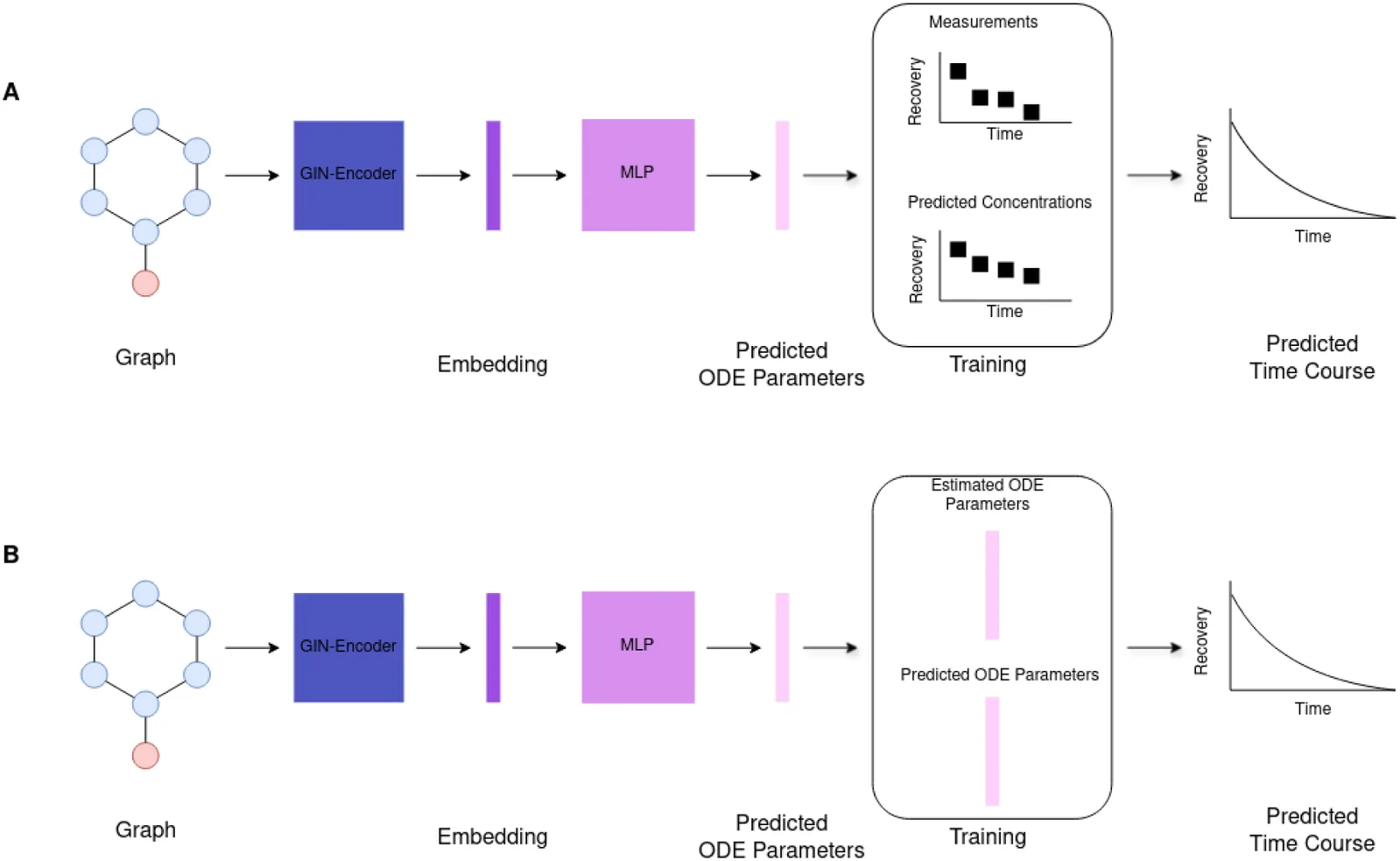
Two modeling approaches were explored as part of this study. (A) Constrained time course: a graph neural network maps molecular and environmental metadata to the parameters of the governing ODEs; integration using the analytical solutions produces concentration–time trajectories, and the model is trained end-to-end against experimental measurements. (B) Conventional approach: the network learns to predict ODE parameters that were pre-estimated by fitting the ODEs to the measured time course; consequently, it does not leverage explicit time-course information during training.

Graph isomorphism networks (GINs^39^) were employed to encode the molecular structure. The GIN encoder output was aggregated via sum pooling, and the resulting representation was passed to a multilayer perceptron (MLP) to estimate the parameters of the selected ODE model. Both the GIN and MLP architectures included batch normalization to improve training stability. The number of hidden channels in the GIN and the number of units in the MLP were treated as hyperparameters and optimized during model selection. To ensure compatibility with the analytical solutions of the ODE systems, the predicted parameters were transformed as follows:

- All predicted rate constants were passed through a ReLU activation to enforce non-negativity.
- The g parameter in the DFOP model was passed through a sigmoid transformation to constrain its value to [0,1].

For each measurement, compound recovery at time t was computed using the transformed predicted parameters, the time point t, and the initial recovery value at t = 0, based on the analytical solution of the selected kinetic model. This formulation converts the training task into a standard regression problem, allowing the use of mean squared error (MSE) as the loss function and treating each time step as an independent sample.

Model optimization was performed using the AdamW optimizer,^40^ with weight decay and learning rate tuned during the hyperparameter search.

For the avoidance of doubt, the modeled target values were the raw, experimentally estimated percentage recovery values, rather than the percentage of the experimentally estimated initial recovery at the start of the experiment (c0), i.e., we did not normalize recovery values by c0. Hence, analytical or chemical property issues that could lead to a deviation in c0 from the theoretical 100% would also affect the modeled target values. When assessing the regression performance of the constrained time-course model, as well as the conventional QSAR approach (see below), this means that providing the model with the experimentally determined c0 will naturally minimize the absolute deviations between the predicted and experimentally observed recovery percentage values. (Indeed, we consider this issue when carrying out prospective validation on the Time-Split Test Set.) Nonetheless, since the models are trained to predict kinetic parameters, the reliability of the model to predict the evolution of the percentage recovery values from c0 will be independent of whether the initial c0 value was provided. Hence, their ability to predict DT_50_ or DT_90_ (defined as the time points at which recovery is 50% or 90% of c0, respectively) will be independent of whether the experimental c0 is supplied.

#### Conventional QSAR Approach: Modeling of Prefitted Kinetic Parameters

The implementation of neural networks in the conventional QSAR approach closely mirrors the architecture used in constrained time-series models. The key distinction lies in the training objective: rather than inserting predicted parameters into ODEs during training, the model directly optimizes the parameters (i.e., rate constants and the fraction of the compound in the fast-degrading compartment for DFOP) based on prior curve-fitting results derived from experimental data (Figure 2B).

As in the constrained setup, rate constants were passed through a ReLU activation to enforce nonnegativity, and the g parameter in the DFOP model was transformed using a sigmoid function to restrict its value to [0,1]. These transformations ensure compatibility with analytical solutions of the kinetic models.

In this framework, each experiment is treated as a single sample (in contrast to the time-series approach, where each time step is considered an individual sample). Model evaluation is performed over the entire predicted time course, allowing an assessment of how well the learned parameters reproduce the observed dissipation dynamics.

#### Hyperparameter Optimization Protocols Performed for Each Soil Metadata Configuration

Hyperparameter tuning for both the constrained time-series models and the conventional QSAR models was performed using Optuna,^41^ with 100 trials per run and no pruning. Hyperparameters were separately tuned for each modeling paradigm and each combination of soil metadata incorporated into the modeling process, including models based purely on molecular structures (i.e., without soil metadata).

Hyperparameter tuning was guided by performance on the validation set, evaluated after each epoch. The model from the best-performing epoch was saved, and its corresponding MSE was used for hyperparameter optimization. Details of the hyperparameter search space (Table S1) and all investigated soil metadata configurations (Table S2) are provided in the Supporting Information.

### Fingerprints Computed for Chemical Space Visualization and Applicability Domain Calculations

Morgan fingerprints were computed using RDKit with a bond radius of 3 and hashed to a bit vector of length 1024. For both chemical space visualization and applicability domain calculations, Tanimoto distances^42–44^ were computed as (1 – Tanimoto similarity) between these molecules.

### Chemical Space Visualization

UMAP^45^ projections of the chemical space were generated using the same hyperparameters as those used by Fooladi et al.^46^ We present plots that include and exclude public-domain structures from the Pesticide Properties Database^47^ (PPDB, version 25–11–03), allowing us to focus on differences in chemical space between the Initial Data Set and the Time-Split Test Set, as well as on structures provided by Syngenta compared to public-domain plant protection product chemistry. To reduce visual clutter and focus on gross trends in the distributions, the distributions of subsets (training set, validation set, test set, time-split test set, and PPDB) of the data set within the UMAP coordinates were represented using kernel density estimation. Full details can be found in the open-source GitHub repository specified in the Data and Software Availability Statement.

### Applicability Domain

Defining an applicability domain (AD) is a critical aspect of modern QSAR modeling.^48^ In this study, a compound is considered within the applicability domain if and only if two conditions are met:

1. Structural Similarity: The compound must be sufficiently similar to those in the Training Set. To assess this, first remove duplicate structures in the Training Set based on stereochemistry-independent InChIs (motivated by the fact that compounds occur multiple times in the Training Set, associated with different studies, soils, and time points, and by the fact that our distance calculations are independent of stereochemistry). Subsequently, an approach similar to that found to work well in recent QSAR studies^49^ was applied: (i) the average Tanimoto distance of each compound in the training set to its *k*-nearest neighbors, denoted as d̅^*j*^, is computed; (ii) the resulting distribution is summarized by its mean and standard deviation; (iii) for a new compound, the mean distance to its k-nearest neighbors, d̅ and a z-score,
8 $$z=\frac{\mathrm{d̅}-\frac{1}{J}\overset{J}{\underset{j}{\sum }}{\mathrm{d̅}}^{j}}{SD\left({\mathrm{d̅}}^{j}\right)}$$
  are computed.
  If the corresponding p-value (assuming a normal distribution) is less than or equal to 0.05, then the compound is considered outside the structural applicability domain. In the current work, the value of k was set to 5.
2. Metadata range compliance: The associated soil metadata must fall within the range observed in the training set. An experiment is considered outside the metadata applicability domain if any numerical metadata feature lies below the minimum or above the maximum value, or if any qualitative value lies outside the set of values encountered during training.

### Full Model Performance Statistics

In addition to the MSE of the individual percentage recoveries (for t > 0), which was minimized during training and hyperparameter optimization, the following performance statistics were computed based on the time-course data: the mean absolute error (MAE) and the coefficient of determination (R^2^).

Finally, performance statistics were computed based on the derived DT_50_ and DT_90_ values. These include classification performance statistics based on the EU persistence trigger of DT_50_ < 120 days,^16^ along with an assessment of the models’ ability to identify low-persistence compounds (DT_50_ < 60 days). The models’ ability to correctly assign the compound to DT_90_ categories using the same thresholds was also assessed. In all cases, when a given set of kinetic ODEs is assumed during the modeling process, the predicted values were compared with those derived from the experimental data under the same assumptions. For the Test Set from the Initial Data Set, these performance statistics are summarized in the Supporting Information.

For the time-split test set, performance statistics were also computed within and outside the applicability domain, since these were not sampled from the same distribution and reflect the gold standard of a real-world application of the model to screen new compounds of interest. In that context, applying an applicability domain assessment to gauge the expected reliability of the predictions is essential.^49^

## Results

We evaluated the models’ performance primarily based on their ability to accurately predict the time course of dissipation. In addition, we report informative case studies and assess the models’ ability to predict whether DT_50_ and DT_90_ occur before or after 60 or 120 days.

During hyperparameter optimization, we selected, for each Modeling Setup, the model with the best validation-set performance. A Modeling Setup is defined by the employed

- metadata features used (including the option of ignoring soil metadata)
- ODE set (SFO, DFOP, SFORB)
- modeling paradigm (constrained time course vs conventional QSAR approach).

We compared the performance of all selected models on the test set, with a focus on the best combination of soil metadata. Finally, the models performing best on the Test Set (i.e., those generated with the best combination of soil metadata) and the models based solely on molecular structure were further evaluated on an external temporal split, i.e., the Time-Split Test Set. Where we refer to the best combination of soil metadata on the Test Set, both when selecting the models to take forward to apply to the Time-Split Test Set and when summarizing the key results in Table 1 and 2, we mean the models with the lowest Test Set MSE.

Before presenting the performance of the optimized models on the Test Set and the Time-Split Test Set, we illustrate the distribution of our data set and all subsets within chemical space.

### Chemical Space Coverage

As shown in Figure 3, the chemical space represented by the Validation Set and the Test Set closely matches that of the Training Set, whereas the chemical space represented by the Time-Split Test Set includes regions that do not overlap with the Training Set distribution. In addition, there is a clear degree of separation between the chemical space covered by the modeled data set, comprising confidential PPP chemicals and lead compounds, and the public-domain PPP compounds represented by the PPDB.

**Figure 3:**
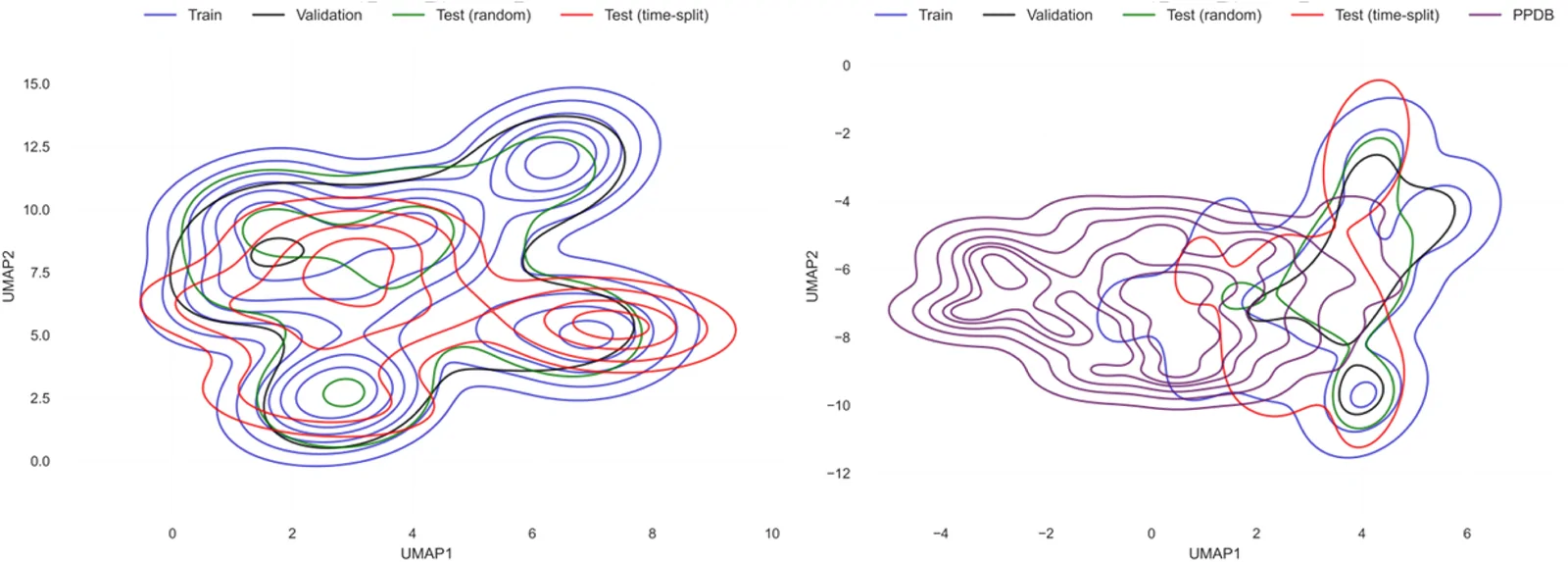
UMAP visualization of the chemical space of the Initial Data Set (comprising the randomly sampled Training Set, Validation Set, and Test Set) and the Time-Split Test Set (left), alongside a comparison with the chemical space of the PPDB (right). To avoid overlapping points obscuring the underlying data distributions, Kernel Density Estimate plots of the point distributions in UMAP coordinates are presented for all subsets. In all plots, structures from different subsets are annotated using consistent colors: Training Set (blue), Validation Set (black), Test Set (green), Time-Split Test Set (red), and PPDB (purple). Note that the UMAP coordinates were computed separately for both panels, i.e., the axes are not directly comparable. This is because the purpose of these plots is to illustrate the relative distribution within chemical space for the different subsets in each plot.

### Model Performance on the Random Test Set: Conventional QSAR vs Constrained Time-Series Approach

Initially, we focused our performance assessment on the models’ ability to predict the evolution of the time-course percentage recovery values from the experimental c0 at the start of the experiment, using the experimental soil metadata. We used the standard regression metrics MSE, MAE, and R^2^.

Following the selection of the best model for each model setup (based on the MSE obtained on the validation set), we tested the selected models on the test set. To account for uneven compound frequencies in the data set, we computed, in addition to the standard performance statistics, the average MSE and MAE per compound on the test set.

Table 1 summarizes the key results of our performance tests. For the sake of clarity, the best-performing combination of soil metadata features is highlighted. The results demonstrate that the constrained time-series approach outperforms the conventional QSAR method.

**Table 1:** Summary of Model Performance on the Test Set^a^

Modeling	Kinetics	Metadata^b^	MSE	MAE	R^2^	Avg.^c^ MSE	Avg. MAE
Conventional	SFO	OM, CEC, pH, Moisture	1097.85	24.70	–0.26	1097.22	24.76
Conventional	DFOP	OM, CEC, pH, Moisture	692.32	21.73	0.20	706.40	21.98
Conventional	SFORB	OM, CEC, pH, Sand, Silt, Clay	994.43	24.17	–0.14	991.28	24.14
Conventional	SFO	-	2059.23	35.21	–1.37	2118.95	35.89
Conventional	DFOP	-	707.15	22.71	0.19	717.69	22.90
Conventional	SFORB	-	1209.62	25.58	–0.39	1213.77	25.65
Constrained	SFO	CEC, pH	477.07	15.09	0.45	485.71	15.35
Constrained	DFOP	OM, Sand, Silt, Clay	478.38	**14.69**	0.45	467.44	**14.52**
Constrained	SFORB	pH, Soil Classification	**471.61**	15.00	**0.46**	**448.74**	14.60
Constrained	SFO	-	602.59	17.40	0.31	620.75	17.64
Constrained	DFOP	-	632.00	18.63	0.27	630.58	18.59
Constrained	SFORB	-	702.00	19.08	0.19	679.56	18.72

a For each metric, the best result is highlighted in bold font. For clarity, only the best results obtained with soil metadata are shown here, whereas the results obtained with all combinations of soil metadata are shown in Figure 4.

b Metadata feature set that yielded the best performance on this test set for the specified mechanistic assumption and modeling approach after optimizing the performance of the model corresponding to any single combination on the validation set. The results obtained for the validation-set-optimized models, with all combinations of soil metadata, are shown in Figure 4.

c Per-compound values were averaged across all compounds in the test set.

The best-performing conventional QSAR model was a DFOP model incorporating metadata (i.e., organic matter, cation exchange capacity, pH (CaCl_2_), and moisture), achieving an R^2^ value of 0.20. The conventional DFOP model without metadata performed slightly worse, with an R^2^ of 0.19. For the conventional SFO and SFORB models, even the best configurations yielded negative R^2^ values, indicating that predictions were less accurate than the mean recoveries.

In contrast, the constrained time-series approach yielded predictive performance that was consistently better than using the mean recovery, with consistently positive R^2^ values that typically exceeded those obtained with the conventional QSAR approach, even when no soil metadata was utilized. (This was consistently the case, when only the best Test Set results are considered.)

On the test set, the constrained models without metadata achieved R^2^ values of 0.31 for SFO, 0.27 for DFOP, and 0.19 for SFORB. Including metadata consistently improved performance across all three differential equation systems, when focusing on the best models identified using the Test Set in Table 1, with the best models reaching R^2^ values of 0.45 for SFO and DFOP, and 0.46 for SFORB.

From these findings, we conclude that the constrained time-series approach performs well across different ODE systems. However, no single ODE system consistently outperformed the other systems. Given that the SFO model effectively captures most dissipation processes and that both DFOP and SFORB are generalizations of SFO, this is not wholly surprising. Nonetheless, DFOP and SFORB offer greater expressive power while remaining mechanistically grounded. Therefore, their use can be justified by their ability to capture biphasic behavior. Moreover, when metadata features were added, the best models performed at least as well as those based on SFO assumptions.

A comparison of all metadata combinations and modeling paradigms (shown in Figure 4 and Table S3) also supports our second conclusion that the inclusion of metadata can increase model performance. However, no single metadata configuration consistently yielded the best results. For the constrained time-series models, Figure 4 shows that performance generally improved when metadata was included, and that the specific choice of metadata was not often critical. In contrast, conventional QSAR-like models showed no clear trend. However, these results should be interpreted with caution, as the performance of the conventional models was generally poor.

**Figure 4:**
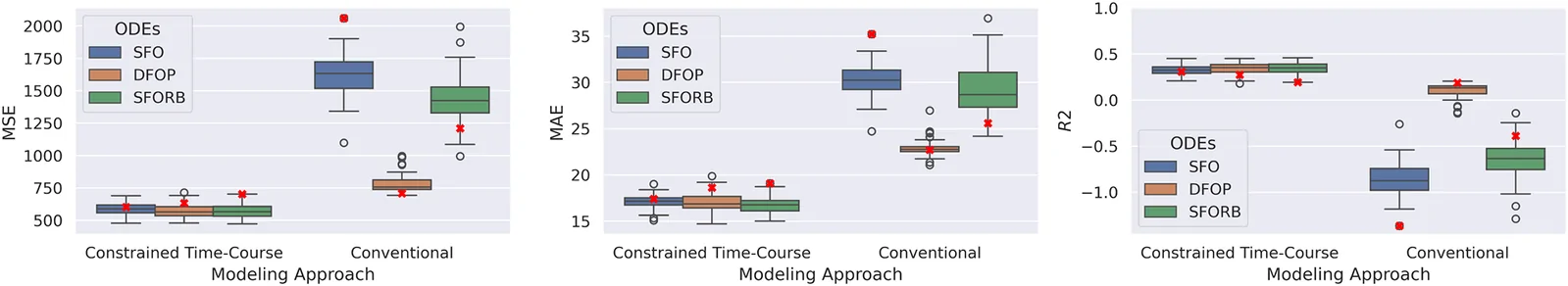
Comparison of model performance on the Test Set across different Modeling Setups. Each point in the boxplot represents the performance of one of the metadata-enhanced models, not just the best results for the Test Set, which are shown in Table 1, along with the corresponding performance statistic value excluding soil metadata superimposed as a red cross.

Finally, it is worth noting that the average per-compound MAE and MSE values, averaged across all compounds in the Test Set (Table 1; Table S3) largely align with the overall performance trends. This suggests that the results are not disproportionately influenced by a small number of outliers or compounds which have been tested more frequently—yielding more time courses—but rather reflect consistent predictive accuracy across the data set.

### DT_50_ and DT_90_ Classification Performance on the Test Set: Constrained Time-Series vs Conventional QSAR Approach

Given the regulatory relevance of soil persistence, we assess whether the models can predict whether a compound’s DT_50_ or DT_90_ exceeds the 60-day or 120-day thresholds. To evaluate this capability, we predicted DT_50_ and DT_90_ values using the parameters derived from both conventional QSAR models and constrained time-series models.

For the constrained time-series models, we compared predictions only with experimental values derived from the percentage recovery values using the same kinetic model (ODE) built into the constrained time-series model. The same consistency was ensured, during the evaluations, between the ODE parameters being predicted to derive the DT50 and DT90 values from the conventional QSAR approach and the ODE used to derive the experimental DT50 and DT90 values. Model performance was evaluated using standard classification metrics: Matthews Correlation Coefficient (MCC), accuracy (ACC), balanced accuracy (BA), and F1-Score—the harmonic mean of recall and precision for the “positive” class, i.e., DT_50_ (or DT_90_) less than the threshold.

Table 2 reports the MCC values obtained on the test set by the best model for each modeling setup. For comparison, the performance of models that disregard metadata is also reported. A comprehensive report of the test-set performance of all investigated models is available in the Supporting Information.

**Table 2:** DT_50_ and DT_90_ Classification Performance of the Best-Performing SFO Regressors on the Test Set. The Performance is Expressed as the Matthews Correlation Coefficient (MCC)^a^

			DT_50_		DT_90_	
Modeling	Kinetics	Metadata	60 d	120 d	60 d	120 d
Conventional	SFO	OM+CEC+pH+Moisture	0.37	0.38	0.39	0.42
Conventional	DFOP	OM+CEC+pH+Moisture	0.16	0.14	0	0
Conventional	SFORB	OM+CEC+pH+Sand+Silt+Clay	0.18	0.20	0.41	0.29
Conventional	SFO	-	0.07	0	–0.11	0
Conventional	DFOP	-	0	0	0	0
Conventional	SFORB	-	0.39	0.35	0.34	0.37
Constrained	SFO	CEC+pH	0.60	0.30	**0.67**	0.62
Constrained	DFOP	OM+Sand+Silt+Clay	0.66	0.62	0.52	0.33
Constrained	SFORB	pH-Soil Classification	**0.70**	**0.63**	0.53	0.51
Constrained	SFO	-	0.61	0.51	0.49	**0.67**
Constrained	DFOP	-	0.60	0.55	0.44	0.33
Constrained	SFORB	-	0.63	0.59	0.30	0.39

a For each metric, the best result is highlighted in a bold font. For the sake of clarity, only the best results obtained with soil metadata are reported. The results obtained with all combinations of soil metadata are shown in Figures 5 and S1.

The classification results obtained on the Test Set were broadly consistent with the regression findings (Table 1). While the conventional QSAR model performed poorly in predicting full time courses, it still achieved modest performance, in some cases, on the simpler classification task. However, the constrained time-series approach outperformed the conventional model across almost all combinations of classification thresholds, metrics, and model types (ODE type—with or without metadata). Nonetheless, the inclusion of soil metadata appeared to improve model performance less consistently. We discuss both points in more depth in the subsequent paragraphs.

For regression predictions (Table 1), the conventional paradigm was consistently outperformed by the constrained time-course approach. This was generally observed, although not always, for the classification predictions. This is illustrated by the fact that the MCC values (Table 2) for DT_50_ and DT_90_ classification at 60 and 120 days almost always improved when moving from the conventional QSAR approach to the constrained time-course approach. (The two exceptions were DT_50_ at 120 days with SFO and soil metadata, and DT_90_ at 60 days with SFORB and no soil metadata. Here, it should also be recalled that Table 2 compares results obtained with the best combinations for soil metadata for this Test Set, which may not be the same for the constrained time-course and conventional approach.) These trends were generally observed when performing direct pairwise comparisons between all models, i.e., comparing results obtained with the same combination of metadata for the MCC and other classification performance statistics (see the Supporting Information). These results suggest that training on the full time course can offer advantages over training solely on parameters of ODEs, even when the primary interest lies in the prediction of the half-life (DT_50_) or DT_90_ (provided that the complete curve is available).

However, unlike the regression results obtained with the best metadata-informed models (Table 1), the classification outcomes did not show as clear a benefit from metadata integration, even in the constrained time-course paradigm. Interestingly, for classification, the constrained time-series model without metadata outperformed its metadata-enhanced counterpart for SFO and DT_50_ classification (threshold of 120 days), whereas the conventional model benefited from metadata inclusion.

Our findings regarding the benefits of metadata inclusion are also evident when moving beyond the best models that harness metadata (Figure 5). However, including metadata can sometimes lead to a notable drop in classification performance (Figures 5 and Figure S1).

**Figure 5:**
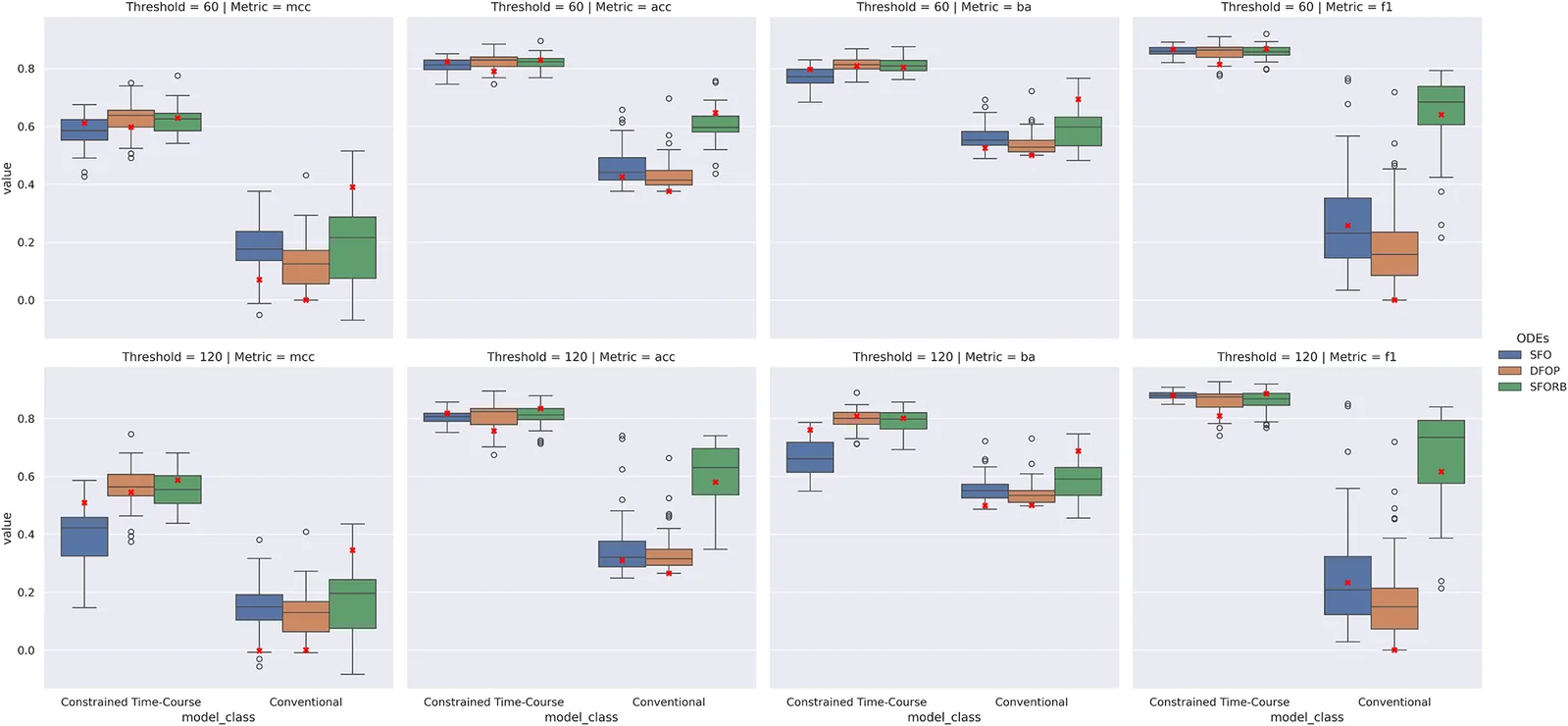
Comparison of DT_50_ classification performance on the Test Set at different thresholds, according to different metrics (shown at the top of each subfigure), across distinct Modeling Setups. In this experiment, the results are independent of c0. The box plots illustrate how different combinations of metadata affect classification outcomes, with the corresponding performance statistic value excluding soil metadata superimposed as a red cross. Here, MCC= Matthews Correlation coefficient; acc = accuracy (total fraction correct); ba = balanced accuracy; f1 = f1-score.

Importantly, our classification setup differs from traditional classification frameworks, in which models are typically trained directly on binned half-life categories.^19,23,49–51^ In our case, we trained regression models for predicting time-course parameters, derived DT_50_ values from these, and evaluated the models’ predictions against two thresholds. Although this approach does not allow for computing class probabilities naturally, compared with some explicit classification algorithms,^49^ it is noteworthy that this approach can be used to assign to any threshold, which can be adjusted post hoc. Furthermore, compared to a multitask classifier, these predictions are guaranteed to be consistent with one another, whereas a naive multitask classifier might simultaneously predict that a compound has a DT_50_ below 60 days and above 120 days.

Another interesting approach, building on the recent work of Salz et al.,^52^ would be to compute point estimates of DT_50_ directly, along with an estimate of the probability distribution around that point, which would allow for post hoc classification with the corresponding probabilities. In principle, our constrained time-course models could be retrained to allow approaches such as deep-learning ensembles or, with changes to the learning process, Bayes via backprop to obtain uncertainty estimates in the modeled kinetic parameters^53^ and, hence, the derivation of probabilistic post hoc classification, e.g., predicting the probability of DT_50_ > 120 days. Another approach would be to adapt our code to apply Monte Carlo dropout during inference^53^ to achieve the same aim. However, we note that, while not universally observed, Monte Carlo dropout has been found to be notably less reliable than strong Bayesian alternatives in various contexts.^53,54^

Finally, we acknowledge that the sometimes very poor performance of the conventional QSAR approach—predicting prefitted parameters—is particularly stark. Indeed, the classification performance for DT_50_ appears no better than random guessing (MCC = 0) in various scenarios for which results are reported in Table 2. Conversely, other recently reported models that have attempted to predict prefitted DT_50_ values directly or to build DT_50_ classifiers according to predefined thresholds using conventional QSAR approaches have reported better results.^49,52^

Indeed, the works published by Salz et al. and Hosni et al. show that conventional regression^52^ or classification^49^ QSAR models trained on SFO DT_50_ values can achieve good performance. However, we think that these performance differences from those of our baseline models can be explained by two major differences. First, although SFO DT_50_ values can be directly transformed into SFO rate constants, the tasks are not necessarily equivalent, and it might be numerically less challenging to train a model on DT_50_ values than on rate constants. However, although it would be possible to first train on SFO half-lives and subsequently predict a time course, it would not be trivial to apply this pattern to biphasic systems. Second, Hosni et al. and Salz et al. used DT_50_ values obtained by aggregating multiple experiments, whereas we did not aggregate by compound. This was necessary because we also utilized metadata as additional features. Since the mentioned studies differ in numerical difficulty and evaluation schemes, it is not possible to directly compare the results.

### Analyses of Predictions for Specific Time-Courses for Individual Compounds Provide Insights beyond Overall Performance Statistics

The statistics reported above for the test set provide valuable insights into the overall performance of the constrained time-course models in predicting how the percentage recovery would evolve based on the initial data point and, where these features were incorporated, the experimentally determined soil metadata for the tested sample. However, deeper insights into model behavior can be obtained by considering case studies of specific time courses for individual compounds selected from that test set.

While metadata commonly improves overall model performance statistics, a closer inspection of individual test cases, particularly those with repeated measurements under different experimental conditions, reveals a more nuanced picture. Figure 6 illustrates this using a compound tested across two different experimental conditions. For each condition, we plotted predictions from two constrained time-course models assuming SFO kinetics: one trained without metadata and another incorporating cation exchange capacity (CEC) and pH (CaCl_2_). The metadata-informed model was selected for its superior performance on the test set.

**Figure 6:**
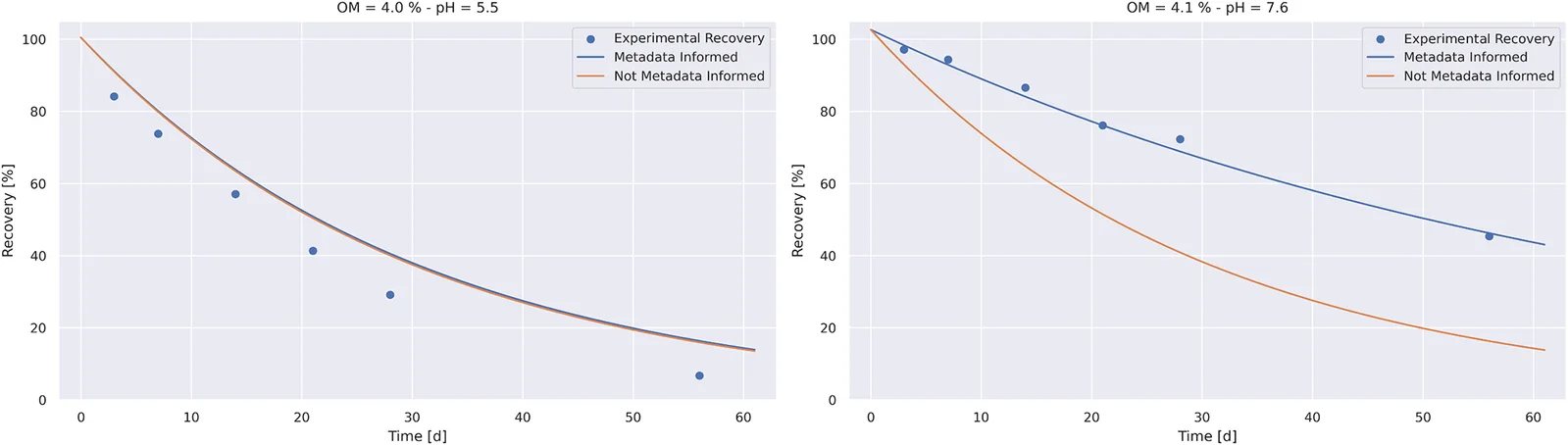
Example predictions from constrained time-course (SFO) models for a compound tested under two experimental conditions, illustrating how the impact of metadata on performance can vary with experimental conditions. Here, the varying conditions corresponded to soil organic matter (OM) percentage and pH. The metadata-informed model considered cation exchange capacity (CEC) and pH as additional features to supplement the molecular graph features learned by the graph neural network molecular architecture, whereas the other model considered the molecular graph only. The metadata-informed model closely matches the observed data in both cases, while the model without metadata shows larger deviations in one example, but not both. This highlights how metadata can substantially improve prediction accuracy in specific cases, but this may depend on the specific conditions under which the compound was tested.

In this example (Figure 6), the metadata-informed model produced highly accurate predictions for a compound tested under two experimental conditions. For the first experimental setup (OM = 4.0%, pH = 5.5), the metadata-informed model achieved a good R^2^ of 0.89 (MAE of 8.45) and an almost perfect R^2^ of 0.99 (MAE of 1.57) for the second example (OM = 4.1%, pH = 7.6). The model without metadata performed slightly better on the first example, with an R^2^ of 0.90 and an MAE of 8.09, but performed poorly on the second example, with an R^2^ of −0.71 and an MAE of 20.62. Consequently, the metadata-informed model performed better across both time courses, i.e., when computing performance statistics based on the percentage recovery values (*t* > 0) for the time courses under both experimental conditions, with an R^2^ of 0.95 and an MAE of 5.01, compared to the model without metadata, which achieved an R^2^ of 0.60 and an MAE of 14.36. This case highlights how metadata can substantially enhance predictive accuracy in specific scenarios, even though their overall impact may vary across the data set.

A counterexample is shown in Figure 7. In this case, the same metadata-informed model shown in Figure 6 tended to underperform the model without metadata, though this outcome was contingent on the experimental conditions. This example illustrates that including metadata does not universally improve performance and may, in some cases, lead to less accurate predictions.

**Figure 7:**
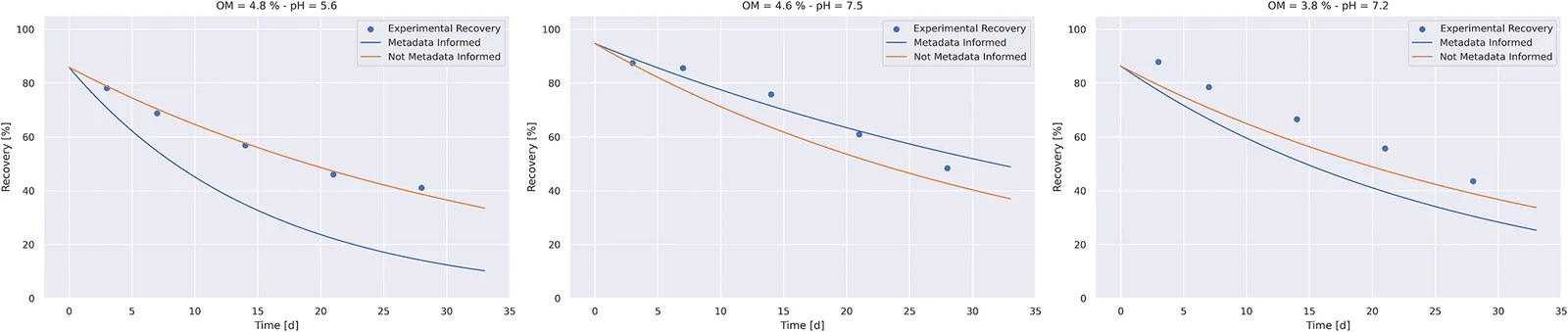
Example predictions for a compound where the same metadata-informed model discussed in Figure 6 underperforms compared to the model without metadata, for three sets of experimental conditions. Despite access to additional input features, the metadata-informed model yields less accurate predictions, highlighting that metadata integration does not universally improve model performance.

The metadata-informed model produced mixed results, with coefficients of determination of −1.13, 0.94, and 0.26 for the three sets of experimental conditions. In contrast, the model without metadata performed better, with coefficients of determination of 0.99, 0.71, and 0.76. Across the three experiments, the metadata-informed models achieved an R^2^ value of 0.20 and an MAE of 11.83. The model without access to metadata outperformed this model, with an R^2^ of 0.83 and an MAE of 5.31.

The examples above raise the question of how estimated model performance may vary as more time courses are generated for test-set compounds across different experimental conditions. To explore this, we analyzed subsets of the test set containing compounds with a minimum number of experiments. The corresponding results are summarized in Table 3.

**Table 3:** Performance of SFO Constrained Time-Course Models with and without Utilization of Metadata for Subsets of the Random Test Set, Depending on the Number of Experiments Available per Compound

		SFO using CEC and pH			SFO not using metadata		
Minimum of Experiments	Number of Compounds	MSE	MAE	R^2^	MSE	MAE	R^2^
1	100	477.07	15.09	0.45	602.59	17.40	0.31
2	31	483.73	15.59	0.49	685.06	18.79	0.28
3	25	517.87	16.22	0.41	732.48	19.42	0.16
4	11	484.34	15.09	0.41	682.08	18.39	0.17
5	8	610.89	17.93	0.31	856.54	22.00	0.03
6	3	808.70	21.59	–0.15	911.62	22.01	–0.30
7	2	675.60	23.81	–0.21	1160.98	26.16	–0.43
8	1	128.00	8.00	0.83	79.49	6.88	0.89

Consistent with the findings in Tables 1, 3 shows that the metadata-informed model generally outperforms the model without metadata across all subsets, except for the subset of eight experiments associated with a single compound, which may be a statistical fluke due to too few data points to obtain robust statistics. As the threshold for the number of experiments increased, the performance tended to decline for both models. However, the decline is more gradual for the meta-data-informed model. Notably, while the MAE remained relatively stable, the MSE increases sharply for the model without metadata, suggesting an increasing number of severe mispredictions. This pattern suggests that the absence of metadata may lead to more frequent outlier errors as data complexity increases. That said, these results should be interpreted with caution, as subset sizes become small when the threshold exceeds four experiments.

Another noteworthy observation, less apparent from standard metrics, is the tendency of the DFOP models to predict plateaus. In the DFOP model without metadata, at least one of the two rate constants was zero across all test set samples. In the best-performing DFOP model with metadata, this behavior was slightly less pronounced but was still present in 143 out of 181 samples.

From a metric perspective, plateau predictions can reduce error by avoiding large deviations. While plateaus are mechanistically plausible within the DFOP and SFORB frameworks, the models appeared to overuse this behavior. Figure 8 illustrates this tendency: both models performed well on the three curves shown, achieving R^2^ values of 0.97 (metadata-informed) and 0.81 (without metadata). However, in this specific case, the plateaus predicted by the models without metadata indicate strong biphasic behavior, although the data indicate that dissipation is well characterized by SFO kinetics, i.e., not biphasic.

**Figure 8:**
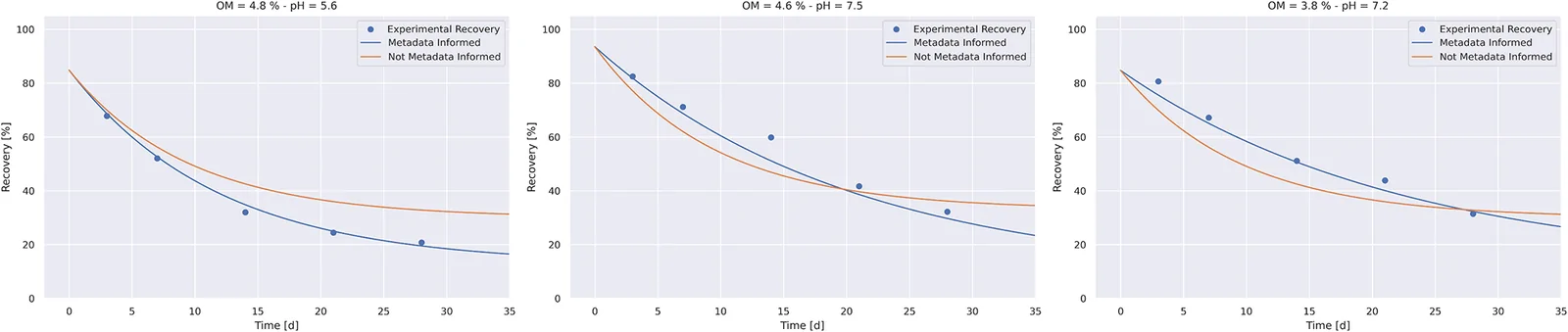
Example predictions from DFOP-constrained time-course models for time-course data generated for the same compound under different experimental conditions illustrating a tendency to overpredict plateau-like behavior. Both the metadata-informed and metadata-free models achieve high coefficients of determination (R^2^ = 0.97 and R^2^ = 0.81, respectively, computed across all time courses), yet both predict biphasic curves with extended plateaus. While such behavior is mechanistically plausible within the DFOP framework, the experimental data could be adequately described using a simpler SFO model, suggesting that the DFOP models may overuse this feature to minimize error. However, our results suggest that including soil metadata somewhat mitigates this tendency, indicating that these models could better anticipate genuine biphasic behavior.

An intriguing possibility, to be considered in future studies, would be to learn which system of ODEs is more useful for specific substances. This could entail characterizing all experimental curves in the data set as biphasic or nonbiphasic and building a machine-learning classifier to predict which chemicals are likely to exhibit biphasic behavior, for which the DFOP or SFORB model would be more suitable to apply than the SFO model.

### Time-Split Results

The predictive performance of the developed models was also assessed using an independent temporal split. Consistent with the random-split Test Set protocol, regression performance was evaluated over the time course, alongside classification performance for DT_50_ and DT_90_, using the same thresholds as above. To ensure the evaluation framework faithfully mirrors real-world applications, where our primary interest is predicting the outcomes of experiments in advance for new compounds of interest in PPP discovery projects, two modifications to the previously considered methodology were explored:

1. Standardization of Initial Concentrations: The initial (t = 0) concentration (c0), i.e., percentage recovery, for each time course was predefined as 100%. This differs from the previously generated results for the test set, where c0 was determined from the experimental values at t = 0, which could yield values below or above 100% due to experimental variability or analytical issues. For cases where c0 was predefined, or the Time-Split Test Set experimental values were used for the time-split results, these are denoted as c0 = 100 and c0 = None, respectively.
2. Imputation of metadata features. Soil-specific metadata features were imputed with the median (numeric features) or the mode (categorical features) for each soil name using the training set. This modification simulates a realistic operational environment in which soil characteristics may be unavailable at the time of prediction, as they depend on the specific soil sample collected once the experiment has been commissioned. For cases where soil metadata was determined from the training set or test set, experimental values were used; the time-split results are denoted as metadata source = train or source = test, respectively.

The two modifications were implemented both independently and in combination to yield four distinct test scenarios. To maintain clarity amid multidimensional performance data, our analysis focuses on R^2^ for regression tasks and MCC for classification. However, all performance statistics are reported in the Supporting Information, and we found that the trends in these statistics were generally consistent with those in the other statistics.

As illustrated in Figure 9 and as can be seen for other metrics in the Supporting Information Excel files, the performance trends observed for the regression models were largely consistent with those observed during the random-split experiments. Specifically, the constrained time-course models outperformed conventional architectures in the vast majority of cases, and integrating soil metadata often yielded a measurable increase in predictive performance. This was seen across all four test scenarios.

**Figure 9:**
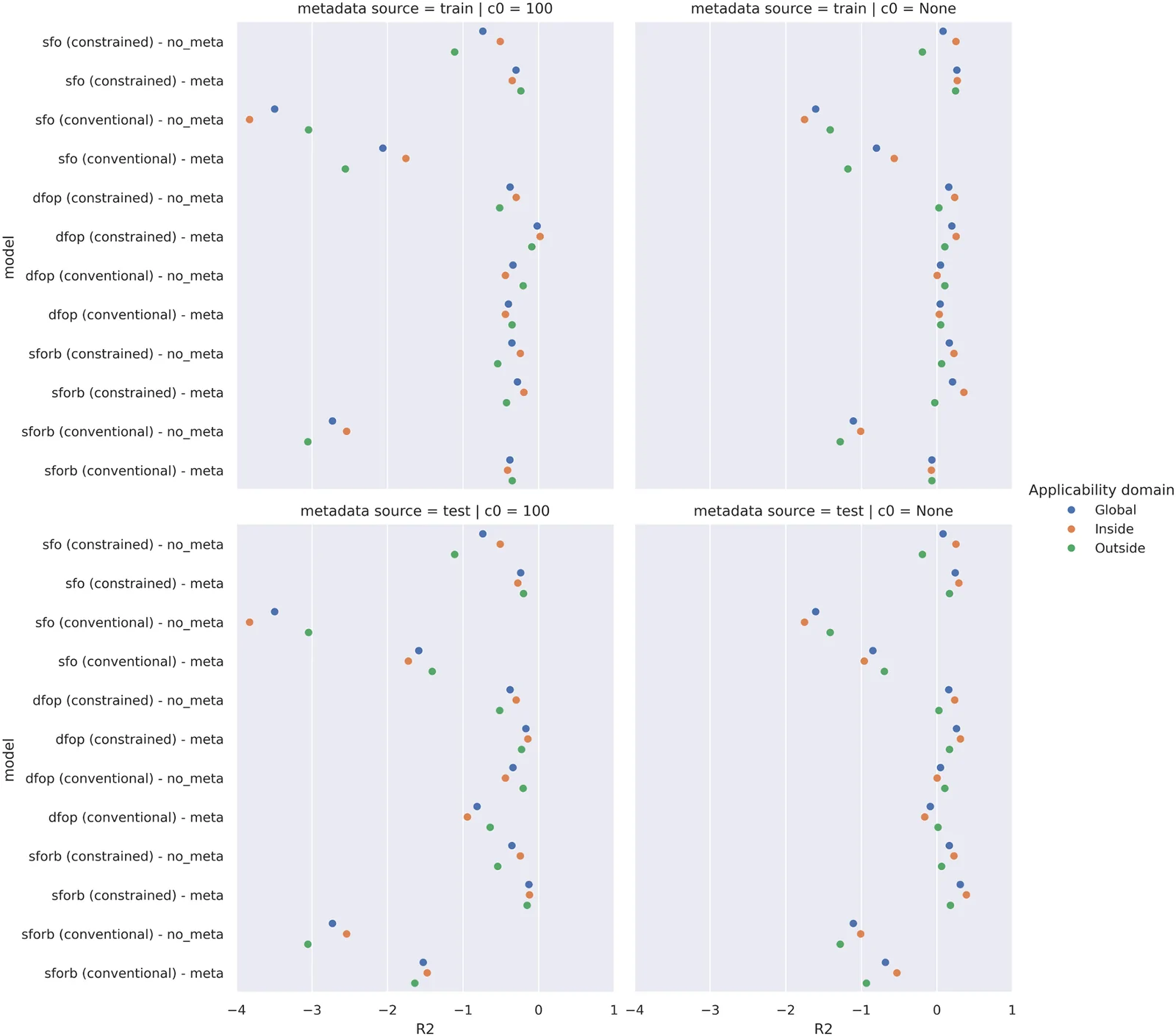
Regression performance on Time-Split Test Set (coefficient of determination, R^2^) comparing the best-performing models (based on the MSE for the random Test Set), with and without access to soil metadata. Here, the use of a predefined vs Time-Split Test Set time-course experimental c0 is denoted using c0 = 100 vs c0 = None, respectively. Likewise, the use of Training Set-imputed soil metadata vs Time-Split Test Set soil-sample experimental metadata is denoted by source = train or source = test, respectively.

The consistency with the trends previously observed for the random test set is especially apparent when considering the results obtained using the time-split test set, experimental initial recovery, and experimental soil metadata to predict the evolution of the time course after t = 0. This setup is equivalent to the evaluation scheme used for the random-split experiments. Considering only models without access to metadata, the best-performing conventional model achieved an R^2^ value of 0.05 (assuming DFOP). In contrast, the worst-performing constrained time course model achieved an R^2^ of 0.08 (assuming SFO), while the best-performing model achieved an R^2^ of 0.17 (assuming SFORB) without metadata. For constrained time-course models, integrating metadata consistently improved the R^2^ values when using the experimental initial recovery (c0) and experimental soil metadata. The coefficient of determination increased from 0.08 to 0.24 for the SFO model, while the SFORB model achieved an R^2^ value of 0.31.

A consistent decline in performance was observed when transitioning from experimentally determined initial recoveries to a hypothetical, and ideal 100% initial recovery. For example, the previously best-performing model (constrained time-course with access to metadata assuming SFORB kinetics) achieved an R^2^ of −0.13, in contrast to 0.31, and was therefore a worse model than the mean of the observed recovery. This decline was expected, since the models were trained to predict the evolution of the soil time-course after t = 0. Hence, even a perfect model would not make good predictions of the absolute percentage recovery values if the experimentally observed c0 value was not supplied, even though it would still predict the shape of the time-course curve.

Interestingly, substituting experimentally observed metadata in the Time-Split Test Set with representative values derived from the Training Set for each soil, less consistently reduced model performance. Exceptions include our observation that, returning to the case where experimental c0 was supplied, the constrained SFO time-course model achieved an R^2^ of 0.24 with observed features, which improved marginally to 0.26 upon the introduction of Training Set-imputed features. In contrast, the constrained SFORB model exhibited substantial performance attenuation under the same conditions. This model’s predictive accuracy fell from an initial R^2^ of 0.31 to 0.21 when using Training Set-imputed metadata features.

The models’ ability to predict absolute percentage recovery values without knowing the experimental c0 (i.e., by setting it to 100%) was poor. However, it should be noted that predicting the shape of the curve is more useful before an experiment than predicting absolute recovery values, which will necessarily be poorly estimated if the initial recovery is unknown and differs from the ideal case of 100%. Moreover, it should be recalled that DT_50_ and DT_90_ correspond to the times at which the recovery falls to 50% and 90% of the recovery observed at t = 0, respectively. For example, if the initial recovery is 90%, DT_50_ would correspond to the time at which the recovery falls to 45%. Hence, reliable predictions of DT_50_ and DT_90_ can still be obtained even if the absolute recovery values are poorly predicted. Indeed, DT_50_ and DT_90_ are invariant to the initial recovery (c0) for models that predict time-course dynamics.

We evaluated the models’ classification performance for DT_50_ and DT_90_ using the previously defined thresholds of 60 and 120 days. In all cases, the same kinetic assumptions (SFO, DFOP, SFORB) used by the models were used to derive the experimental DT_50_ and DT_90_ values to which the predictions were compared. Consistent with the regression results, the constrained time-series approach almost always outperformed conventional architectures across both end points and all thresholds. Considering the MCC values, the only exception to this trend was the MCC obtained outside the domain for the classification of DT_90_ values at 60 days, assuming SFORB kinetics, using the Training Set-imputed experimental metadata. The DT_50_ and DT_90_ classification performance measured as MCC is visualized in Figures 10 and 11, respectively. Comparable plots showing ACC, BA, and F1 score for both classification tasks are provided in the (Supporting Information Figures S2 to S7).

**Figure 10:**
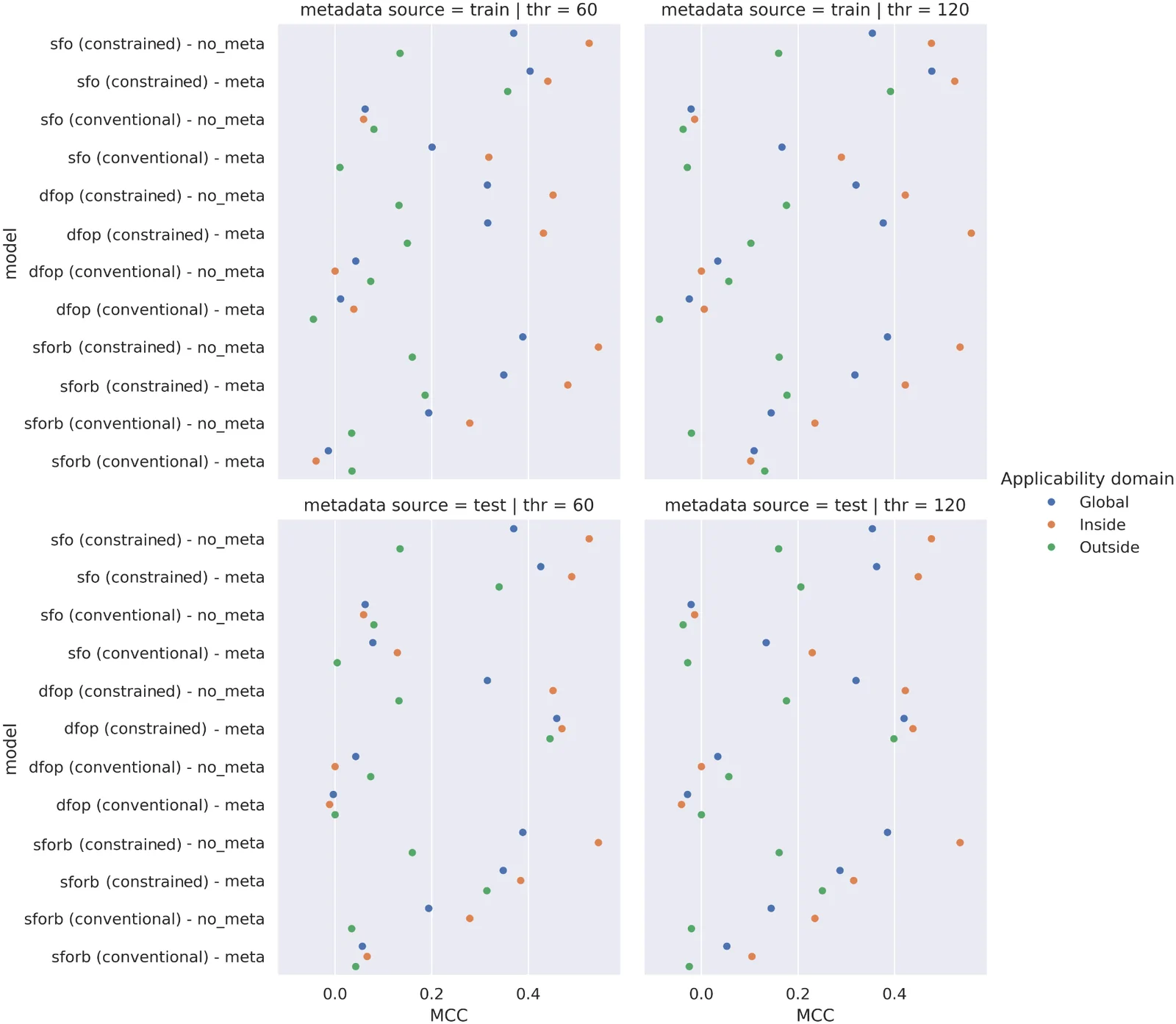
DT_50_ Classification performance on Time-Split Test Set (MCC), comparing the best performing models (based on the MSE for the Test Set), with and without access to soil metadata. Here, the results were independent of c0. Likewise, the use of Training Set imputed soil metadata vs Time-Split Test Set soil sample experimental metadata is denoted using metadata source = train or source = test, respectively.

**Figure 11:**
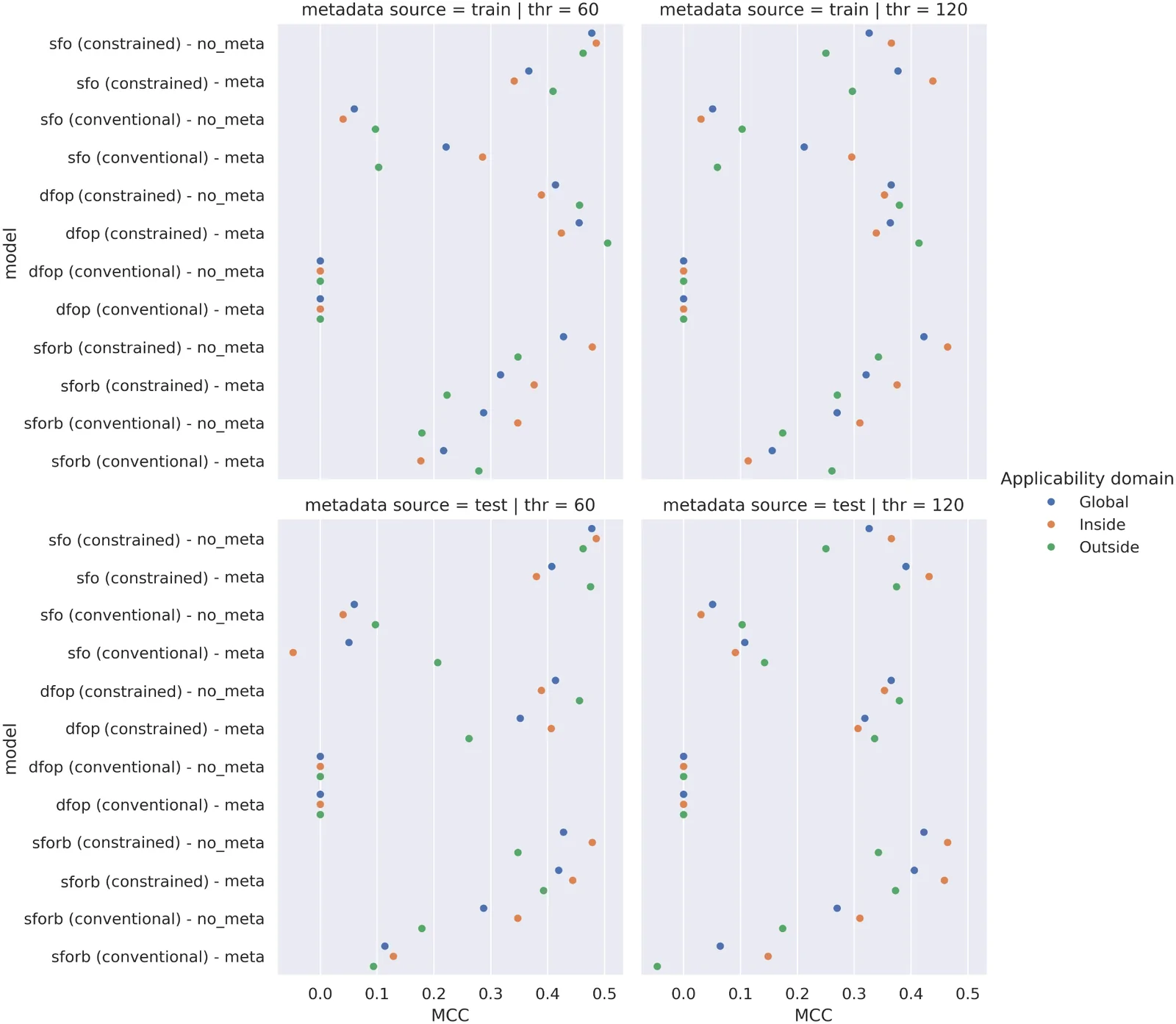
DT_90_ Classification performance on Time-Split Test Set (Matthews Correlation Coefficient, MCC) comparing the best-performing models (based on the MSE for the Test Set), with and without access to soil metadata. Here, the results were independent of c0. The use of Training Set-imputed soil metadata vs Time-Split Test Set soil-sample experimental metadata is denoted using metadata source = train or source = test, respectively.

The constrained time-course models achieved robust predictive power, maintaining MCC values above 0.3 in nearly all scenarios (excluding statistics generated for time courses from outside the domain). The sole exception was the SFORB model using time-split test-set experimental metadata (DT_50_, 120-day threshold), which yielded an MCC of 0.29.

For these constrained time-course models, the impact of incorporating soil metadata on DT_50_ and DT_90_ classification performance, as well as the impact of switching from the Time-Split Test Set experimental to the Training Set-imputed soil metadata, was mixed. In less than two-thirds of the corresponding results with the constrained time-course models, the MCC decreased by at least 0.05. Indeed, in nine out of 36 cases (DT_50_ or DT_90_ x 60 or 120 days x global or inside or outside the domain x SFO, or DFOP or SFORB), the MCC improved by at least 0.05. However, specific sensitivities were observed, including a decrease in the SFORB model’s DT_90_ classification performance at the 60-day threshold, from a global MCC of 0.42 (metadata source = “test”) to 0.32 (metadata source = “train”) upon introducing imputed metadata.

## Conclusion

In this study, we developed and explored a constrained time-series approach for predicting the dissipation kinetics of small molecules in soil from molecular structure and compared it with conventional QSAR modeling. The results show that the constrained time-series model outperforms the conventional QSAR approach across most performance metrics computed for both the randomly sampled (test set) and time-split (time-split test set) test sets. This generally holds true for time-course regression and for classifying DT_50_ and DT_90_ at two thresholds (60 and 120 days). For example, on the randomly sampled test set, the best-performing constrained time-course models achieved an R^2^ of 0.46 when experimentally measured soil metadata was used to augment the predicted time-course evolution from the experimentally measured initial recovery, compared with a best result of 0.31 when only compound structural information was used. In contrast, the best-performing conventional model achieved an R^2^ value of 0.20. Similarly, the highest observed MCC for the DT_50_ (60 days) classification was 0.70, achieved with a constrained time-course model. The best-performing classification performance achieved by a conventional model for this task was an MCC of 0.39.

In contrast to previous studies modeling soil dissipation, we also investigated the impact of different systems of ordinary differential equations (ODEs): single-first-order (SFO), double-first-order-in-parallel (DFOP), and single-first-order-reversible-binding (SFORB). We conclude that, in our case, these did not yield consistent, substantial performance improvements. For example, all three constrained time-course models achieved coefficients of determination of 0.45 or 0.46 on the randomly sampled test set when metadata was utilized. However, since such systems remain mechanistically grounded, their use can be justified for modeling dissipation kinetics in soil when the end-user is interested in assessing the potential for biphasic behavior.

The complexity of modeling dissipation kinetics in soil stems from the inherent complexity of soil as a system. To address this, we incorporated metadata representing soil sample characteristics, such as pH, into the model. Although including metadata does not guarantee improved performance, it can yield meaningful gains. Indeed, for the constrained time-course DFOP and SFORB models, performance on the randomly sampled test set, in terms of R^2^, increased from 0.27 to 0.45 and from 0.19 to 0.46, respectively.

Furthermore, we demonstrated that unlike unconstrained time-series models that may predict unphysical behavior, the constrained approach inherently adheres to mechanistic assumptions by design. This adherence is important even when standard performance statistics might insidiously indicate comparable performance.

To further assess the models’ reliability, we evaluated their ability in predicting a Time-Split Test Set comprising time courses generated for previously unseen compounds tested in studies reported after the initial data set was assembled. Although performance decreased, the constrained time-course models still made meaningful predictions of the time-course evolution from the initial experimental recovery using experimental soil metadata. The best-performing model, which incorporated soil metadata and assumed SFORB kinetics, achieved an R^2^ of 0.31.

Finally, to simulate the application of the models to predict the dissipation time course for new compounds prior to the studies being carried out, the performance on the Time-Split Test Set was updated based on a predefined initial recovery value and soil metadata values imputed from Training Set statistics (in practice, neither of these would be known until a specific sample of soil is collected and the initial measurement of recovery is made at the start of the experiment). In those cases, while poor performance was obtained in predicting the absolute recovery values, the dissipation dynamics were still reasonably well predicted. This is reflected in the fact that the constrained time course models maintained an MCC of at least 0.29 across all investigated classification problems and models. In some cases, the performance reached an MCC of 0.48 or even 0.56 when only compounds and experiments that were calculated to lie inside the applicability domain were considered. Nonetheless, the observed decrease in performance underscores the need to build models that explicitly account for uncertainty in soil metadata.

Further improvements might be possible by trying to better capture sorption processes in the modeling process. One way to do that might involve using the difference between measured and theoretical (100% recovery) initial concentrations (c0) during training. However, careful consideration would need to be given to how to incorporate this into predictions made in advance of starting a soil dissipation study, since this difference would, by definition, not be known a priori. Nonetheless, it might be able to predict this from the molecular structure.

Ultimately, we have demonstrated that our introduction of a mechanistically constrained time-course framework improves the state of the art for predicting the dissipation of small molecules in soil. Including soil metadata can, in some cases, enhance the predictions.

## Supplementary Material







## Data Availability

All code used for chemical space exploration, model building, and model evaluation has been released on GitHub under the MIT open-source license (https://github.com/VincentAlexanderScholz/soil-dissipation-kinetics). The version of the code corresponding to the results reported in this publication has been made available as a GitHub release (https://github.com/VincentAlexanderScholz/soil-dissipation-kinetics/releases/tag/paper). The code repo includes a full description of all software dependencies, installation instructions, and guidance on reproducing the results in this manuscript. This includes a full description of the data set format, so that researchers who collect comparable data can organize their data in the same fashion and build and evaluate models using the open-source code. The Syngenta data sets referenced in this work are not publicly available due to their proprietary, commercially sensitive nature.

## Notes

Classification results of all models on the Test Set (XLSX)
Document describing the SFORB analytical solution used by the constrained time-course models, soil metadata fields and combinations considered for modeling, and additional performance statistics tables and figures mentioned in the main text (PDF)
Time-Split Test Set results (XLSX)
