# Supplementary material for: Modeling the Soil Dissipation Kinetics of Small Molecules
Source: J Chem Inf Model. 2026 Oct 28;66(21):13809–26. doi: 10.1021/acs.jcim.6c01812 (PMC13753509; doi:10.1021/acs.jcim.6c01812)
Supplement: Supplementary file 1 [file ci6c01812_si_002.pdf]

Supporting Information

Modelling the Soil Dissipation Kinetics of Small Molecules

Vincent-Alexander Scholz,[1,2] Richard Marchese Robinson,[3] Sevil Payvandi,[3] Steffen Hirte,[1,2] Timothy J.C. O'Riordan,[3*] Johannes Kirchmair[1*]

1. Department of Pharmaceutical Sciences, University of Vienna, Josef-Holaubek-Platz 2, 1090, Austria
2. Vienna Doctoral School of Pharmaceutical, Nutritional and Sport Sciences (PhaNuSpo), University of Vienna, 1090 Vienna, Austria
3. Syngenta Crop Protection, Jealott's Hill International Research Centre, Bracknell, Berkshire, RG42 6EY United Kingdom

[*] Corresponding authors: tim.o_riordan@syngenta.com; johannes.kirchmair@univie.ac.at

Analytical Solution of the Single-First-Order Reversible Binding model

The analytical solution of the Single-First-Order Reversible Binding (SFORB) model is more complex than those of SFO and DFOP. As detailed by reference[1], for the biphasic models, the total amount of compound A is given by

$$[A] = [A_1] + [A_2] = [A_1]_0 \left(\frac{k_{12}+k_{21}-b_1}{b_2-b_1} e^{-b_1 t} + \frac{k_{12}+k_{21}-b_2}{b_1-b_2} e^{-b_2 t} \right).$$

Where b_1 and b_2 are given by:

$$b_1 = \frac{1}{2}(k_{12} + k_{21} + k_1) + \sqrt{\frac{1}{4}(k_{12} + k_{21} + k_1)^2 + k_{12}k_{21} - (k_{12} + k_1)k_{21}}$$

and

$$b_2 = \frac{1}{2}(k_{12} + k_{21} + k_1) - \sqrt{\frac{1}{4}(k_{12} + k_{21} + k_1)^2 + k_{12}k_{21} - (k_{12} + k_1)k_{21}}.$$

Hyperparameter Optimization

Hyperparameter optimisation was performed using Optuna.[2] The search space for all hyperparameters is summarised in Table S1. The same search space was applied to both the conventional models and the constrained time series models. All 49 metadata combinations used are summarised in Table S2.

Table S1: Search space of the hyperparameters for the GNNs used for the constrained time series approach and the conventional approach.

Parameter	Search Space
Hidden channels during convolution	[16, 128]
Number of convolutional layers	[1, 4]
Units of GIN MLP	[32, 256]
Number of linear layers	[1, 4]
Activation Function	{ReLU, ELU, LeakyReLU}
Learning Rate	[0.00001, 0.01]
Weight Decay	[0.0001, 0.10]

Table S2: List of all metadata feature combinations. Note: all references to pH refer to pH measured in CaCl2

Index	Metadata features used
1	No metadata
2	Soil (name of the soil used)
3	Organic Matter
4	Cation Exchange Capacity
5	pH
6	Moisture
7	Sand, Silt, Clay
8	Organic Matter, Cation Exchange Capacity
9	Organic Matter, pH
10	Organic Matter, Moisture
11	Organic Matter, Sand, Silt, Clay
12	Cation Exchange Capacity, pH
13	Cation Exchange Capacity, Moisture
14	Cation Exchange Capacity, Sand, Silt, Clay
15	pH, Moisture
16	pH, Sand, Silt, Clay
17	Moisture, Sand, Silt, Clay
18	Organic Matter, Cation Exchange Capacity, pH
19	Organic Matter, Cation Exchange Capacity, Moisture
20	Organic Matter, Cation Exchange Capacity, Sand, Silt, Clay
21	Organic Matter, pH, Moisture
22	Organic Matter, pH, Sand, Silt, Clay
23	Organic Matter, Moisture, Sand, Silt, Clay
24	Cation Exchange Capacity, pH, Moisture
25	Cation Exchange Capacity, pH, Sand, Silt, Clay
26	Cation Exchange Capacity, Moisture, Sand, Silt, Clay
27	pH, Moisture, Sand, Silt, Clay
28	Organic Matter, Cation Exchange Capacity, pH, Moisture
29	Organic Matter, Cation Exchange Capacity, pH, Sand, Silt, Clay
30	Organic Matter, Cation Exchange Capacity, Moisture, Sand, Silt, Clay
31	Organic Matter, pH, Moisture, Sand, Silt, Clay
32	Cation Exchange Capacity, pH, Moisture, Sand, Silt, Clay
33	Organic Matter, Cation Exchange Capacity, pH, Moisture, Sand, Silt, Clay
34	Soil Classification
35	Organic Matter, Soil Classification
36	Cation Exchange Capacity, Soil Classification
37	pH, Soil Classification
38	Moisture, Soil Classification
39	Organic Matter, Cation Exchange Capacity, Soil Classification
40	Organic Matter, pH, Soil Classification
41	Organic Matter, Moisture, Soil Classification
42	Cation Exchange Capacity, pH, Soil Classification
43	Cation Exchange Capacity, Moisture, Soil Classification
44	pH, Moisture, Soil Classification
45	Organic Matter, Cation Exchange Capacity, pH, Soil Classification
46	Organic Matter, Cation Exchange Capacity, Moisture, Soil Classification
47	Organic Matter, pH, Moisture, Soil Classification
48	Cation Exchange Capacity, pH, Moisture, Soil Classification
49	Organic Matter, Cation Exchange Capacity, pH, Moisture, Soil Classification

Performance Metrics

Table S3: Performance on the test set (random split) for each metadata feature set.

Modeling	Kinetics	Metadata	MSE	MAE	R^2	Avg. MSE	Avg. MAE
Constrained	SFO	no_meta	602.59	17.40	0.31	620.75	17.64
Constrained	SFO	soil	608.82	17.57	0.30	599.14	17.48
Constrained	SFO	om	540.74	17.05	0.38	537.13	17.15
Constrained	SFO	cec	562.29	17.32	0.35	548.78	17.23
Constrained	SFO	ph_cacl2	614.40	17.87	0.29	620.83	18.00
Constrained	SFO	moisture	494.23	15.64	0.43	478.64	15.46
Constrained	SFO	sand-silt-clay	635.26	17.35	0.27	630.91	17.33
Constrained	SFO	om-cec	674.47	18.19	0.23	668.87	18.18
Constrained	SFO	om-ph_cacl2	657.09	18.40	0.25	650.27	18.41
Constrained	SFO	om-moisture	601.51	17.07	0.31	587.11	16.89
Constrained	SFO	om-sand-silt-clay	605.76	16.37	0.30	562.03	15.90
Constrained	SFO	cec-ph_cacl2	477.07	15.09	0.45	485.71	15.35
Constrained	SFO	cec-moisture	615.58	17.15	0.29	617.00	17.19
Constrained	SFO	cec-sand-silt-clay	633.16	17.52	0.27	657.44	17.87
Constrained	SFO	ph_cacl2-moisture	613.47	16.80	0.30	599.27	16.64
Constrained	SFO	ph_cacl2-sand-silt-clay	568.65	16.41	0.35	556.31	16.35
Constrained	SFO	moisture-sand-silt-clay	640.37	17.54	0.26	636.50	17.55
Constrained	SFO	om-cec-ph_cacl2	626.34	17.73	0.28	622.91	17.75
Constrained	SFO	om-cec-moisture	564.68	16.14	0.35	553.75	15.99
Constrained	SFO	om-cec-sand-silt-clay	516.56	15.80	0.41	518.78	15.96
Constrained	SFO	om-ph_cacl2-moisture	514.81	16.34	0.41	514.54	16.37
Constrained	SFO	om-ph_cacl2-sand-silt-clay	674.43	19.00	0.23	664.41	18.97
Constrained	SFO	om-moisture-sand-silt-clay	503.86	15.33	0.42	499.12	15.34
Constrained	SFO	cec-ph_cacl2-moisture	654.00	17.95	0.25	655.61	18.08
Constrained	SFO	cec-ph_cacl2-sand-silt-clay	647.60	17.52	0.26	661.18	17.78
Constrained	SFO	cec-moisture-sand-silt-clay	557.88	17.22	0.36	548.97	17.15
Constrained	SFO	ph_cacl2-moisture-sand-silt-clay	583.46	18.08	0.33	585.58	18.15
Constrained	SFO	om-cec-ph_cacl2-moisture	580.17	17.52	0.33	566.71	17.35
Constrained	SFO	om-cec-ph_cacl2-sand-silt-clay	529.53	16.11	0.39	528.74	16.08
Constrained	SFO	om-cec-moisture-sand-silt-clay	594.89	16.73	0.32	579.88	16.62
Constrained	SFO	om-ph_cacl2-moisture-sand-silt-clay	616.98	17.40	0.29	606.53	17.39
Constrained	SFO	cec-ph_cacl2-moisture-sand-silt-clay	596.88	16.95	0.31	588.72	16.84
Constrained	SFO	om-cec-ph_cacl2-moisture-sand-silt-clay	584.85	17.02	0.33	592.17	17.09
Constrained	SFO	soil_classification	587.83	17.64	0.32	584.84	17.58
Constrained	SFO	om-soil_classification	587.12	17.27	0.33	588.85	17.41
Constrained	SFO	cec-soil_classification	566.67	16.76	0.35	565.00	16.84
Constrained	SFO	ph_cacl2-soil_classification	690.08	18.09	0.21	693.53	18.22
Constrained	SFO	moisture-soil_classification	522.94	16.11	0.40	537.33	16.38
Constrained	SFO	om-cec-soil_classification	665.48	17.41	0.24	662.31	17.42
Constrained	SFO	om-ph_cacl2-soil_classification	556.14	16.74	0.36	554.45	16.76
Constrained	SFO	om-moisture-soil_classification	567.91	16.95	0.35	555.77	16.92
Constrained	SFO	cec-ph_cacl2-soil_classification	546.19	16.73	0.37	545.76	16.77
Constrained	SFO	cec-moisture-soil_classification	598.70	17.18	0.31	614.37	17.49
Constrained	SFO	ph_cacl2-moisture-soil_classification	583.05	17.12	0.33	577.68	17.17
Constrained	SFO	om-cec-ph_cacl2-soil_classification	585.54	16.84	0.33	588.06	16.99
Constrained	SFO	om-cec-moisture-soil_classification	596.84	17.18	0.31	585.92	17.08
Constrained	SFO	om-ph_cacl2-moisture-soil_classification	543.09	16.53	0.38	546.41	16.63
Constrained	SFO	cec-ph_cacl2-moisture-soil_classification	632.13	17.51	0.27	643.68	17.75
Constrained	SFO	om-cec-ph_cacl2-moisture-soil_classification	522.03	16.24	0.40	526.00	16.39
Constrained	DFOP	no_meta	632.00	18.63	0.27	630.58	18.59
Constrained	DFOP	soil	612.45	17.60	0.30	603.78	17.58
Constrained	DFOP	om	569.48	17.35	0.35	570.88	17.47
Constrained	DFOP	cec	538.27	16.38	0.38	548.53	16.57
Constrained	DFOP	ph_cacl2	585.83	17.82	0.33	583.20	17.85
Constrained	DFOP	moisture	564.45	16.44	0.35	545.08	16.28
Constrained	DFOP	sand-silt-clay	532.69	16.67	0.39	547.34	16.83
Constrained	DFOP	om-cec	612.61	17.77	0.30	616.29	17.87
Constrained	DFOP	om-ph_cacl2	587.17	16.54	0.33	604.41	16.83
Constrained	DFOP	om-moisture	623.85	18.04	0.28	632.18	18.30
Constrained	DFOP	om-sand-silt-clay	478.38	14.69	0.45	467.44	14.52
Constrained	DFOP	cec-ph_cacl2	604.20	18.21	0.31	602.24	18.32
Constrained	DFOP	cec-moisture	494.94	15.16	0.43	510.63	15.40
Constrained	DFOP	cec-sand-silt-clay	713.78	19.87	0.18	735.54	20.15

Constrained	DFOP	ph_cacl2-moisture	623.83	17.51	0.28	635.77	17.73
Constrained	DFOP	ph_cacl2-sand-silt-clay	508.62	16.23	0.42	490.16	16.04
Constrained	DFOP	moisture-sand-silt-clay	508.19	15.58	0.42	509.34	15.68
Constrained	DFOP	om-cec-ph_cacl2	543.06	16.30	0.38	531.53	16.23
Constrained	DFOP	om-cec-moisture	624.41	18.64	0.28	632.62	18.80
Constrained	DFOP	om-cec-sand-silt-clay	534.88	16.45	0.39	520.03	16.24
Constrained	DFOP	om-ph_cacl2-moisture	539.60	16.69	0.38	547.45	16.92
Constrained	DFOP	om-ph_cacl2-sand-silt-clay	601.12	17.08	0.31	605.45	17.20
Constrained	DFOP	om-moisture-sand-silt-clay	492.11	15.51	0.43	479.01	15.40
Constrained	DFOP	cec-ph_cacl2-moisture	558.47	16.88	0.36	558.54	16.94
Constrained	DFOP	cec-ph_cacl2-sand-silt-clay	511.98	16.79	0.41	514.42	16.89
Constrained	DFOP	cec-moisture-sand-silt-clay	534.73	15.66	0.39	539.38	15.76
Constrained	DFOP	ph_cacl2-moisture-sand-silt-clay	549.10	17.29	0.37	551.83	17.39
Constrained	DFOP	om-cec-ph_cacl2-moisture	529.76	17.19	0.39	519.41	17.13
Constrained	DFOP	om-cec-ph_cacl2-sand-silt-clay	521.99	15.61	0.40	518.08	15.66
Constrained	DFOP	om-cec-moisture-sand-silt-clay	585.49	16.44	0.33	588.53	16.39
Constrained	DFOP	om-ph_cacl2-moisture-sand-silt-clay	690.69	19.20	0.21	682.61	19.22
Constrained	DFOP	cec-ph_cacl2-moisture-sand-silt-clay	559.18	16.64	0.36	547.02	16.52
Constrained	DFOP	om-cec-ph_cacl2-moisture-sand-silt-clay	568.60	17.66	0.35	566.65	17.69
Constrained	DFOP	soil_classification	564.22	17.03	0.35	576.15	17.14
Constrained	DFOP	om-soil_classification	574.71	16.80	0.34	564.63	16.72
Constrained	DFOP	cec-soil_classification	605.53	17.26	0.30	624.90	17.50
Constrained	DFOP	ph_cacl2-soil_classification	598.01	16.78	0.31	599.91	16.82
Constrained	DFOP	moisture-soil_classification	533.67	17.38	0.39	542.79	17.48
Constrained	DFOP	om-cec-soil_classification	603.52	16.74	0.31	601.30	16.64
Constrained	DFOP	om-ph_cacl2-soil_classification	610.72	18.38	0.30	614.22	18.53
Constrained	DFOP	om-moisture-soil_classification	548.88	17.20	0.37	544.31	17.25
Constrained	DFOP	cec-ph_cacl2-soil_classification	670.18	17.84	0.23	674.89	17.98
Constrained	DFOP	cec-moisture-soil_classification	667.16	18.13	0.23	684.16	18.37
Constrained	DFOP	ph_cacl2-moisture-soil_classification	548.24	16.74	0.37	560.84	16.99
Constrained	DFOP	om-cec-ph_cacl2-soil_classification	653.66	18.24	0.25	680.19	18.61
Constrained	DFOP	om-cec-moisture-soil_classification	526.23	16.07	0.40	514.95	15.98
Constrained	DFOP	om-ph_cacl2-moisture-soil_classification	544.36	15.70	0.37	528.28	15.41
Constrained	DFOP	cec-ph_cacl2-moisture-soil_classification	559.92	16.42	0.36	563.68	16.49
Constrained	DFOP	om-cec-ph_cacl2-moisture-soil_classification	544.64	17.13	0.37	529.08	16.97
Constrained	SFORB	no_meta	702.00	19.08	0.19	679.56	18.72
Constrained	SFORB	soil	565.58	16.29	0.35	580.21	16.52
Constrained	SFORB	om	519.89	16.27	0.40	533.02	16.60
Constrained	SFORB	cec	545.86	16.10	0.37	555.81	16.30
Constrained	SFORB	ph_cacl2	569.12	16.93	0.35	553.28	16.80
Constrained	SFORB	moisture	649.11	17.89	0.25	663.41	18.19
Constrained	SFORB	sand-silt-clay	501.63	15.18	0.42	496.58	15.14
Constrained	SFORB	om-cec	562.24	16.17	0.35	541.18	15.86
Constrained	SFORB	om-ph_cacl2	638.79	17.89	0.27	655.61	18.17
Constrained	SFORB	om-moisture	585.72	17.92	0.33	586.28	18.07
Constrained	SFORB	om-sand-silt-clay	549.37	15.90	0.37	535.35	15.79
Constrained	SFORB	cec-ph_cacl2	597.42	16.42	0.31	585.09	16.27
Constrained	SFORB	cec-moisture	569.64	16.91	0.35	555.26	16.68
Constrained	SFORB	cec-sand-silt-clay	566.55	17.24	0.35	582.03	17.48
Constrained	SFORB	ph_cacl2-moisture	531.26	16.37	0.39	536.41	16.55
Constrained	SFORB	ph_cacl2-sand-silt-clay	525.60	16.06	0.40	526.15	16.18
Constrained	SFORB	moisture-sand-silt-clay	657.74	18.39	0.24	662.83	18.45
Constrained	SFORB	om-cec-ph_cacl2	536.33	15.75	0.38	527.88	15.57
Constrained	SFORB	om-cec-moisture	594.15	16.86	0.32	600.46	17.02
Constrained	SFORB	om-cec-sand-silt-clay	522.39	15.79	0.40	515.04	15.75
Constrained	SFORB	om-ph_cacl2-moisture	538.97	16.96	0.38	540.58	17.10
Constrained	SFORB	om-ph_cacl2-sand-silt-clay	516.46	15.96	0.41	504.61	15.89
Constrained	SFORB	om-moisture-sand-silt-clay	501.93	15.78	0.42	500.87	15.77
Constrained	SFORB	cec-ph_cacl2-moisture	635.96	18.09	0.27	642.33	18.24
Constrained	SFORB	cec-ph_cacl2-sand-silt-clay	540.25	16.20	0.38	540.99	16.31
Constrained	SFORB	cec-moisture-sand-silt-clay	552.55	16.44	0.37	552.28	16.44
Constrained	SFORB	ph_cacl2-moisture-sand-silt-clay	558.99	16.91	0.36	566.13	17.00
Constrained	SFORB	om-cec-ph_cacl2-moisture	518.57	15.45	0.40	509.23	15.40
Constrained	SFORB	om-cec-ph_cacl2-sand-silt-clay	647.17	17.10	0.26	654.81	17.31
Constrained	SFORB	om-cec-moisture-sand-silt-clay	606.11	16.85	0.30	591.35	16.72
Constrained	SFORB	om-ph_cacl2-moisture-sand-silt-clay	658.54	18.72	0.24	643.79	18.60
Constrained	SFORB	cec-ph_cacl2-moisture-sand-silt-clay	574.18	16.85	0.34	552.10	16.44
Constrained	SFORB	om-cec-ph_cacl2-moisture-sand-silt-clay	633.72	18.51	0.27	635.81	18.65
Constrained	SFORB	soil_classification	542.80	16.37	0.38	569.28	16.67

Constrained	SFORB	om-soil_classification	529.05	16.52	0.39	519.44	16.46
Constrained	SFORB	cec-soil_classification	590.50	17.23	0.32	603.14	17.42
Constrained	SFORB	ph_cacl2-soil_classification	471.61	15.00	0.46	448.74	14.60
Constrained	SFORB	moisture-soil_classification	504.61	16.48	0.42	510.15	16.65
Constrained	SFORB	om-cec-soil_classification	608.99	16.97	0.30	614.69	17.11
Constrained	SFORB	om-ph_cacl2-soil_classification	649.29	17.79	0.25	646.27	17.77
Constrained	SFORB	om-moisture-soil_classification	551.34	15.91	0.37	579.85	16.41
Constrained	SFORB	cec-ph_cacl2-soil_classification	622.91	17.42	0.28	646.46	17.71
Constrained	SFORB	cec-moisture-soil_classification	675.35	17.84	0.22	702.43	18.20
Constrained	SFORB	ph_cacl2-moisture-soil_classification	517.54	15.86	0.41	525.76	15.99
Constrained	SFORB	om-cec-ph_cacl2-soil_classification	577.87	16.74	0.34	555.20	16.40
Constrained	SFORB	om-cec-moisture-soil_classification	500.67	16.11	0.42	514.93	16.28
Constrained	SFORB	om-ph_cacl2-moisture-soil_classification	546.94	16.91	0.37	551.26	17.07
Constrained	SFORB	cec-ph_cacl2-moisture-soil_classification	588.34	17.12	0.32	582.80	17.09
Constrained	SFORB	om-cec-ph_cacl2-moisture-soil_classification	581.13	16.75	0.33	589.65	16.93
Conventional	SFO	no_meta	2059.23	35.21	-1.37	2118.95	35.89
Conventional	SFO	soil	1759.55	31.71	-1.02	1821.34	32.41
Conventional	SFO	om	1620.32	30.23	-0.86	1684.17	30.92
Conventional	SFO	cec	1782.65	31.62	-1.05	1826.55	32.12
Conventional	SFO	ph_cacl2	1722.65	31.38	-0.98	1776.18	31.96
Conventional	SFO	moisture	1456.57	29.03	-0.67	1475.47	29.32
Conventional	SFO	sand-silt-clay	1900.86	33.38	-1.18	1921.36	33.65
Conventional	SFO	om-cec	1421.80	28.23	-0.63	1453.60	28.69
Conventional	SFO	om-ph_cacl2	1591.08	29.58	-0.83	1622.35	29.93
Conventional	SFO	om-moisture	1793.42	31.75	-1.06	1836.19	32.25
Conventional	SFO	om-sand-silt-clay	1807.26	31.97	-1.08	1845.04	32.40
Conventional	SFO	cec-ph_cacl2	1704.98	30.92	-0.96	1776.46	31.72
Conventional	SFO	cec-moisture	1539.32	29.27	-0.77	1544.06	29.22
Conventional	SFO	cec-sand-silt-clay	1638.59	29.92	-0.88	1663.65	30.08
Conventional	SFO	ph_cacl2-moisture	1793.36	31.89	-1.06	1833.57	32.37
Conventional	SFO	ph_cacl2-sand-silt-clay	1696.23	30.73	-0.95	1769.57	31.54
Conventional	SFO	moisture-sand-silt-clay	1632.55	29.79	-0.88	1686.43	30.39
Conventional	SFO	om-cec-ph_cacl2	1678.03	30.60	-0.93	1717.42	31.01
Conventional	SFO	om-cec-moisture	1361.21	27.26	-0.56	1395.49	27.75
Conventional	SFO	om-cec-sand-silt-clay	1517.03	32.23	-0.74	1523.05	32.31
Conventional	SFO	om-ph_cacl2-moisture	1605.51	29.91	-0.84	1678.02	30.77
Conventional	SFO	om-ph_cacl2-sand-silt-clay	1603.14	30.03	-0.84	1667.38	30.76
Conventional	SFO	om-moisture-sand-silt-clay	1626.98	30.13	-0.87	1653.68	30.43
Conventional	SFO	cec-ph_cacl2-moisture	1526.20	32.40	-0.75	1506.81	32.17
Conventional	SFO	cec-ph_cacl2-sand-silt-clay	1340.96	27.09	-0.54	1359.52	27.41
Conventional	SFO	cec-moisture-sand-silt-clay	1744.02	30.93	-1.00	1800.14	31.70
Conventional	SFO	ph_cacl2-moisture-sand-silt-clay	1746.76	31.33	-1.01	1801.99	31.96
Conventional	SFO	om-cec-ph_cacl2-moisture	1097.85	24.70	-0.26	1097.22	24.76
Conventional	SFO	om-cec-ph_cacl2-sand-silt-clay	1416.28	27.83	-0.63	1427.76	28.01
Conventional	SFO	om-cec-moisture-sand-silt-clay	1665	30.47	-0.91	1731.84	31.25
Conventional	SFO	om-ph_cacl2-moisture-sand-silt-clay	1443.60	28.13	-0.66	1473.52	28.50
Conventional	SFO	cec-ph_cacl2-moisture-sand-silt-clay	1606.85	29.77	-0.85	1665.86	30.42
Conventional	SFO	om-cec-ph_cacl2-moisture-sand-silt-clay	1660.62	30.27	-0.91	1714.36	30.91
Conventional	SFO	soil_classification	1787.23	31.74	-1.05	1807.77	32.03
Conventional	SFO	om-soil_classification	1495.21	29.00	-0.72	1558.98	29.73
Conventional	SFO	cec-soil_classification	1506.95	29.22	-0.73	1531.91	29.53
Conventional	SFO	ph_cacl2-soil_classification	1541.12	29.14	-0.77	1618.18	29.99
Conventional	SFO	moisture-soil_classification	1796.35	31.91	-1.06	1843.80	32.45
Conventional	SFO	om-cec-soil_classification	1725.62	30.87	-0.98	1758.32	31.23
Conventional	SFO	om-ph_cacl2-soil_classification	1557.08	29.55	-0.79	1583.08	29.82
Conventional	SFO	om-moisture-soil_classification	1653.82	30.29	-0.90	1701.37	30.84
Conventional	SFO	cec-ph_cacl2-soil_classification	1650.35	29.99	-0.90	1688.37	30.47
Conventional	SFO	cec-moisture-soil_classification	1667.70	30.55	-0.92	1719.92	31.17
Conventional	SFO	ph_cacl2-moisture-soil_classification	1431.73	28.09	-0.64	1484.44	28.76
Conventional	SFO	om-cec-ph_cacl2-soil_classification	1492.43	28.59	-0.71	1479.96	28.31
Conventional	SFO	om-cec-moisture-soil_classification	1657.08	30.26	-0.90	1700.83	30.76
Conventional	SFO	om-ph_cacl2-moisture-soil_classification	1710.77	30.89	-0.97	1774.68	31.62
Conventional	SFO	cec-ph_cacl2-moisture-soil_classification	1514.14	28.82	-0.74	1521.35	28.91
Conventional	SFO	om-cec-ph_cacl2-moisture-soil_classification	1575.01	29.52	-0.81	1624.83	30.10
Conventional	DFOP	no_meta	707.15	22.71	0.19	717.69	22.90
Conventional	DFOP	soil	993.57	26.96	-0.14	980.81	26.85
Conventional	DFOP	om	868.98	23.22	0.00	887.45	23.47
Conventional	DFOP	cec	983.70	24.49	-0.13	1038.43	25.11
Conventional	DFOP	ph_cacl2	774.03	23.14	0.11	763.66	23.19

Conventional	DFOP	moisture	790.11	22.65	0.09	829.25	23.12
Conventional	DFOP	sand-silt-clay	822.15	23.45	0.06	833.52	23.50
Conventional	DFOP	om-cec	777.29	22.53	0.11	792.42	22.77
Conventional	DFOP	om-ph_cacl2	719.78	22.71	0.17	730.36	22.89
Conventional	DFOP	om-moisture	699.95	21.35	0.20	726.79	21.67
Conventional	DFOP	om-sand-silt-clay	739.16	22.49	0.15	751.57	22.68
Conventional	DFOP	cec-ph_cacl2	805.02	22.91	0.08	822.21	23.15
Conventional	DFOP	cec-moisture	748.13	22.97	0.14	772.31	23.31
Conventional	DFOP	cec-sand-silt-clay	927.11	24.08	-0.07	951.95	24.33
Conventional	DFOP	ph_cacl2-moisture	796.69	22.86	0.08	821.16	23.20
Conventional	DFOP	ph_cacl2-sand-silt-clay	756.37	22.84	0.13	777.91	23.14
Conventional	DFOP	moisture-sand-silt-clay	703.91	21.77	0.19	730.92	22.13
Conventional	DFOP	om-cec-ph_cacl2	803.63	22.80	0.08	824.47	23.09
Conventional	DFOP	om-cec-moisture	736.84	22.66	0.15	759.16	22.98
Conventional	DFOP	om-cec-sand-silt-clay	797.35	22.55	0.08	827.33	22.94
Conventional	DFOP	om-ph_cacl2-moisture	712.03	22.23	0.18	733.09	22.53
Conventional	DFOP	om-ph_cacl2-sand-silt-clay	738.24	23.18	0.15	753.44	23.41
Conventional	DFOP	om-moisture-sand-silt-clay	730.05	22.64	0.16	746.40	22.89
Conventional	DFOP	cec-ph_cacl2-moisture	738.72	22.45	0.15	758.51	22.76
Conventional	DFOP	cec-ph_cacl2-sand-silt-clay	790.60	23.01	0.09	803.26	23.25
Conventional	DFOP	cec-moisture-sand-silt-clay	935.70	24.66	-0.07	978.05	25.17
Conventional	DFOP	ph_cacl2-moisture-sand-silt-clay	871.45	22.91	0.00	904.24	23.39
Conventional	DFOP	om-cec-ph_cacl2-moisture	692.32	21.73	0.20	706.40	21.98
Conventional	DFOP	om-cec-ph_cacl2-sand-silt-clay	995.94	24.68	-0.14	1015.03	24.91
Conventional	DFOP	om-cec-moisture-sand-silt-clay	750.98	22.76	0.14	771.78	23.09
Conventional	DFOP	om-ph_cacl2-moisture-sand-silt-clay	737.30	22.98	0.15	754.78	23.22
Conventional	DFOP	cec-ph_cacl2-moisture-sand-silt-clay	736.33	22.76	0.15	755.93	23.05
Conventional	DFOP	om-cec-ph_cacl2-moisture-sand-silt-clay	717.68	22.52	0.18	729.81	22.73
Conventional	DFOP	soil_classification	738.97	22.62	0.15	758.52	22.95
Conventional	DFOP	om-soil_classification	744.62	22.67	0.14	760.67	22.90
Conventional	DFOP	cec-soil_classification	817.02	23.15	0.06	843.73	23.52
Conventional	DFOP	ph_cacl2-soil_classification	792.62	23.06	0.09	818.56	23.42
Conventional	DFOP	moisture-soil_classification	810.90	23.01	0.07	834.78	23.38
Conventional	DFOP	om-cec-soil_classification	758.03	22.64	0.13	786.43	23.02
Conventional	DFOP	om-ph_cacl2-soil_classification	742.17	22.52	0.15	758.12	22.73
Conventional	DFOP	om-moisture-soil_classification	754.33	21.96	0.13	769.12	22.22
Conventional	DFOP	cec-ph_cacl2-soil_classification	734.01	22.31	0.16	758.42	22.65
Conventional	DFOP	cec-moisture-soil_classification	836.84	22.77	0.04	815.54	22.79
Conventional	DFOP	ph_cacl2-moisture-soil_classification	770.87	22.45	0.11	782.25	22.65
Conventional	DFOP	om-cec-ph_cacl2-soil_classification	715.70	22.41	0.18	721.86	22.52
Conventional	DFOP	om-cec-moisture-soil_classification	818.91	23.79	0.06	848.78	24.18
Conventional	DFOP	om-ph_cacl2-moisture-soil_classification	743.75	21.06	0.15	756.66	21.34
Conventional	DFOP	cec-ph_cacl2-moisture-soil_classification	745.45	22.89	0.14	774.72	23.28
Conventional	DFOP	om-cec-ph_cacl2-moisture-soil_classification	821.39	24.09	0.06	831.04	24.25
Conventional	SFORB	no_meta	1209.62	25.58	-0.39	1213.77	25.65
Conventional	SFORB	soil	1279.78	26.46	-0.47	1323.35	26.97
Conventional	SFORB	om	1236.08	26.14	-0.42	1220.80	25.91
Conventional	SFORB	cec	1146.30	25.41	-0.32	1154.06	25.52
Conventional	SFORB	ph_cacl2	1575.72	29.49	-0.81	1618.04	29.98
Conventional	SFORB	moisture	1758.24	34.67	-1.02	1738.43	34.49
Conventional	SFORB	sand-silt-clay	1263.74	27.47	-0.45	1253.37	27.38
Conventional	SFORB	om-cec	1565.81	31.15	-0.80	1598.54	31.62
Conventional	SFORB	om-ph_cacl2	1385.11	29.12	-0.59	1365.62	29.03
Conventional	SFORB	om-moisture	1085.01	24.61	-0.25	1083.06	24.65
Conventional	SFORB	om-sand-silt-clay	1493.23	28.29	-0.72	1557.15	29.07
Conventional	SFORB	cec-ph_cacl2	1720.00	31.63	-0.98	1783.49	32.32
Conventional	SFORB	cec-moisture	1328.44	28.00	-0.53	1325.12	27.91
Conventional	SFORB	cec-sand-silt-clay	1260.70	26.52	-0.45	1244.57	26.30
Conventional	SFORB	ph_cacl2-moisture	1571.70	32.48	-0.81	1527.29	32.12
Conventional	SFORB	ph_cacl2-sand-silt-clay	1400.35	28.66	-0.61	1403.65	28.75
Conventional	SFORB	moisture-sand-silt-clay	1484.37	29.16	-0.71	1504.78	29.45
Conventional	SFORB	om-cec-ph_cacl2	1330.32	26.65	-0.53	1377.54	27.21
Conventional	SFORB	om-cec-moisture	1365.76	27.22	-0.57	1415.84	27.88
Conventional	SFORB	om-cec-sand-silt-clay	1367.65	27.87	-0.57	1376.14	28.00
Conventional	SFORB	om-ph_cacl2-moisture	1335.08	28.09	-0.53	1285.72	27.56
Conventional	SFORB	om-ph_cacl2-sand-silt-clay	1582.38	32.56	-0.82	1583.77	32.63
Conventional	SFORB	om-moisture-sand-silt-clay	1448.75	30.06	-0.66	1380.65	29.31
Conventional	SFORB	cec-ph_cacl2-moisture	1425.92	28.38	-0.64	1442.81	28.62
Conventional	SFORB	cec-ph_cacl2-sand-silt-clay	1454.36	27.95	-0.67	1461.92	28.06

Conventional	SFORB	cec-moisture-sand-silt-clay	1498.74	30.86	-0.72	1479.10	30.62
Conventional	SFORB	ph_cacl2-moisture-sand-silt-clay	1527.80	31.14	-0.76	1488.41	30.77
Conventional	SFORB	om-cec-ph_cacl2-moisture	1289.11	27.33	-0.48	1241.37	26.66
Conventional	SFORB	om-cec-ph_cacl2-sand-silt-clay	994.43	24.17	-0.14	991.28	24.14
Conventional	SFORB	om-cec-moisture-sand-silt-clay	1241.93	27.08	-0.43	1248.86	27.09
Conventional	SFORB	om-ph_cacl2-moisture-sand-silt-clay	1344.57	29.52	-0.54	1324.02	29.24
Conventional	SFORB	cec-ph_cacl2-moisture-sand-silt-clay	1243.91	26.67	-0.43	1239.41	26.65
Conventional	SFORB	om-cec-ph_cacl2-moisture-sand-silt-clay	1642.70	30.64	-0.89	1655.31	30.75
Conventional	SFORB	soil_classification	1993.05	36.93	-1.29	1948.00	36.55
Conventional	SFORB	om-soil_classification	1397.30	28.51	-0.61	1432.30	28.94
Conventional	SFORB	cec-soil_classification	1588.03	29.90	-0.82	1628.56	30.40
Conventional	SFORB	ph_cacl2-soil_classification	1464.38	29.03	-0.68	1464.91	28.99
Conventional	SFORB	moisture-soil_classification	1489.39	31.68	-0.71	1476.74	31.60
Conventional	SFORB	om-cec-soil_classification	1872.92	35.11	-1.15	1861.18	35.03
Conventional	SFORB	om-ph_cacl2-soil_classification	1530.59	31.38	-0.76	1507.72	31.16
Conventional	SFORB	om-moisture-soil_classification	1362.57	29.75	-0.57	1356.90	29.81
Conventional	SFORB	cec-ph_cacl2-soil_classification	1421.19	28.68	-0.63	1414.16	28.55
Conventional	SFORB	cec-moisture-soil_classification	1578.99	32.54	-0.81	1566.89	32.38
Conventional	SFORB	ph_cacl2-moisture-soil_classification	1521.60	31.19	-0.75	1495.53	30.93
Conventional	SFORB	om-cec-ph_cacl2-soil_classification	1180.07	26.94	-0.36	1184.13	27.02
Conventional	SFORB	om-cec-moisture-soil_classification	1441.95	31.07	-0.66	1443.18	31.17
Conventional	SFORB	om-ph_cacl2-moisture-soil_classification	1422.75	28.59	-0.63	1376.29	28.18
Conventional	SFORB	cec-ph_cacl2-moisture-soil_classification	1486.76	30.09	-0.71	1451.38	29.76
Conventional	SFORB	om-cec-ph_cacl2-moisture-soil_classification	1356.50	28.09	-0.56	1355.17	28.16

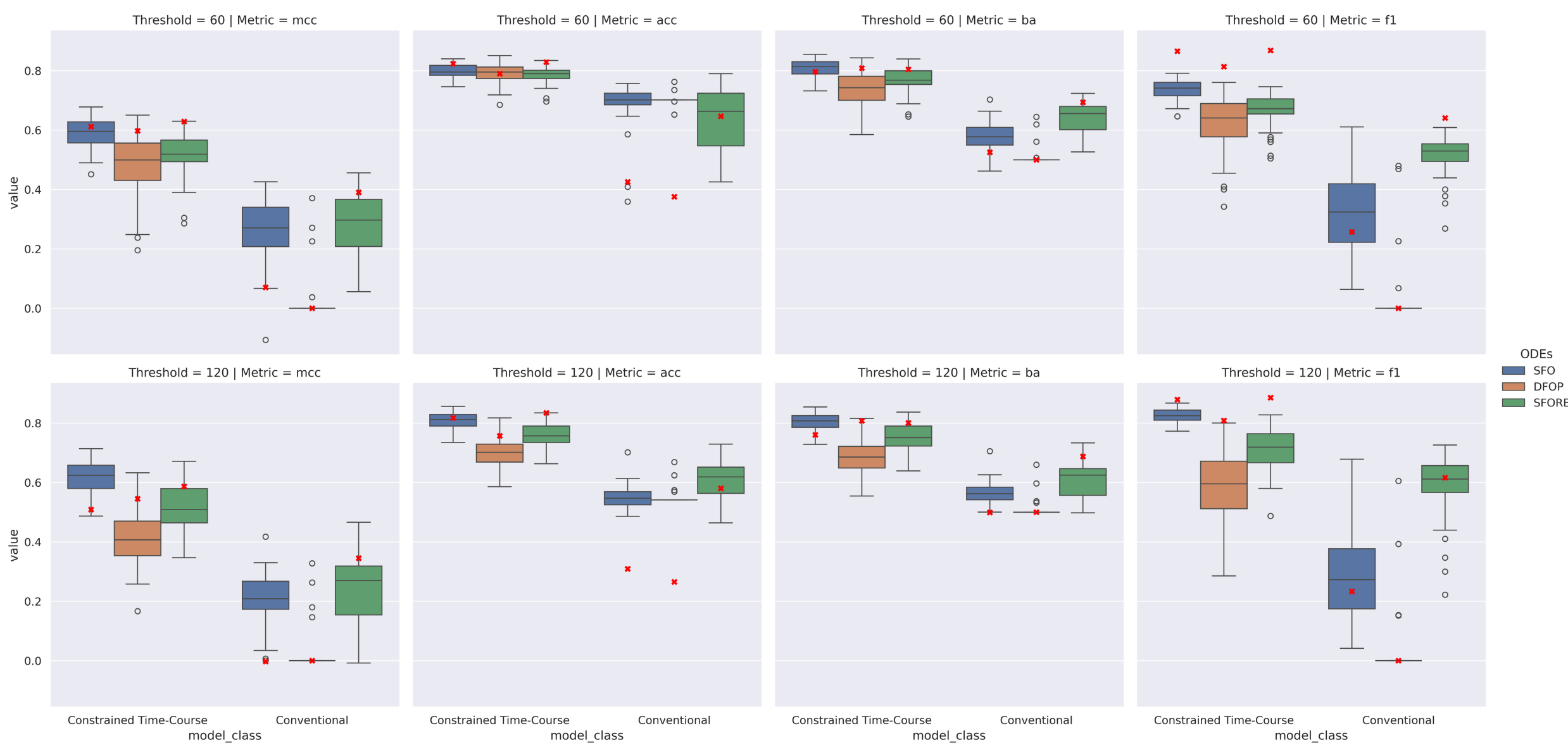

Figure S1: Comparison of DT_{90} classification performance on the random Test Set at different thresholds, according to different metrics (shown at the top of each sub-figure), across modelling approaches, mechanistic assumptions, and metadata configurations. Here, the results were independent of c0. The box plots illustrate how different combinations of metadata affect classification outcomes, with the corresponding performance statistic value, excluding any soil metadata, superimposed as a red cross. Here, MCC= Matthews Correlation coefficient, acc = accuracy (total fraction correct), ba = balanced accuracy, f1 = f1-score

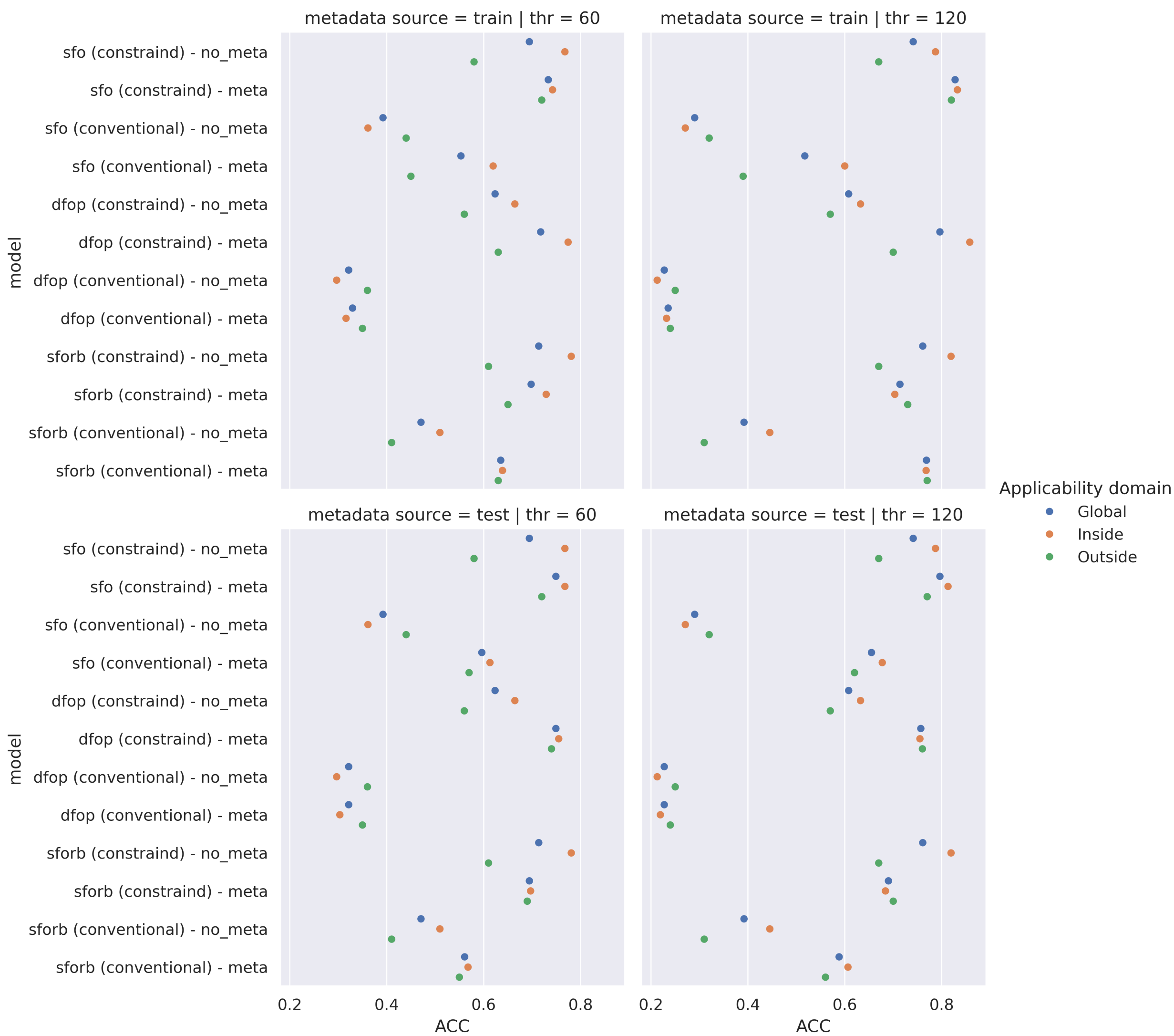

Figure S2: DT_{50} Classification performance on Time-Split Test Set (Accuracy, ACC), comparing the best performing models (based on the MSE for the Test Set), with and without access to soil metadata. Here, the results were independent of c0. Likewise, the use of Training Set imputed soil metadata vs Time-Split Test Set soil sample experimental metadata is denoted using metadata source = train vs test, respectively.

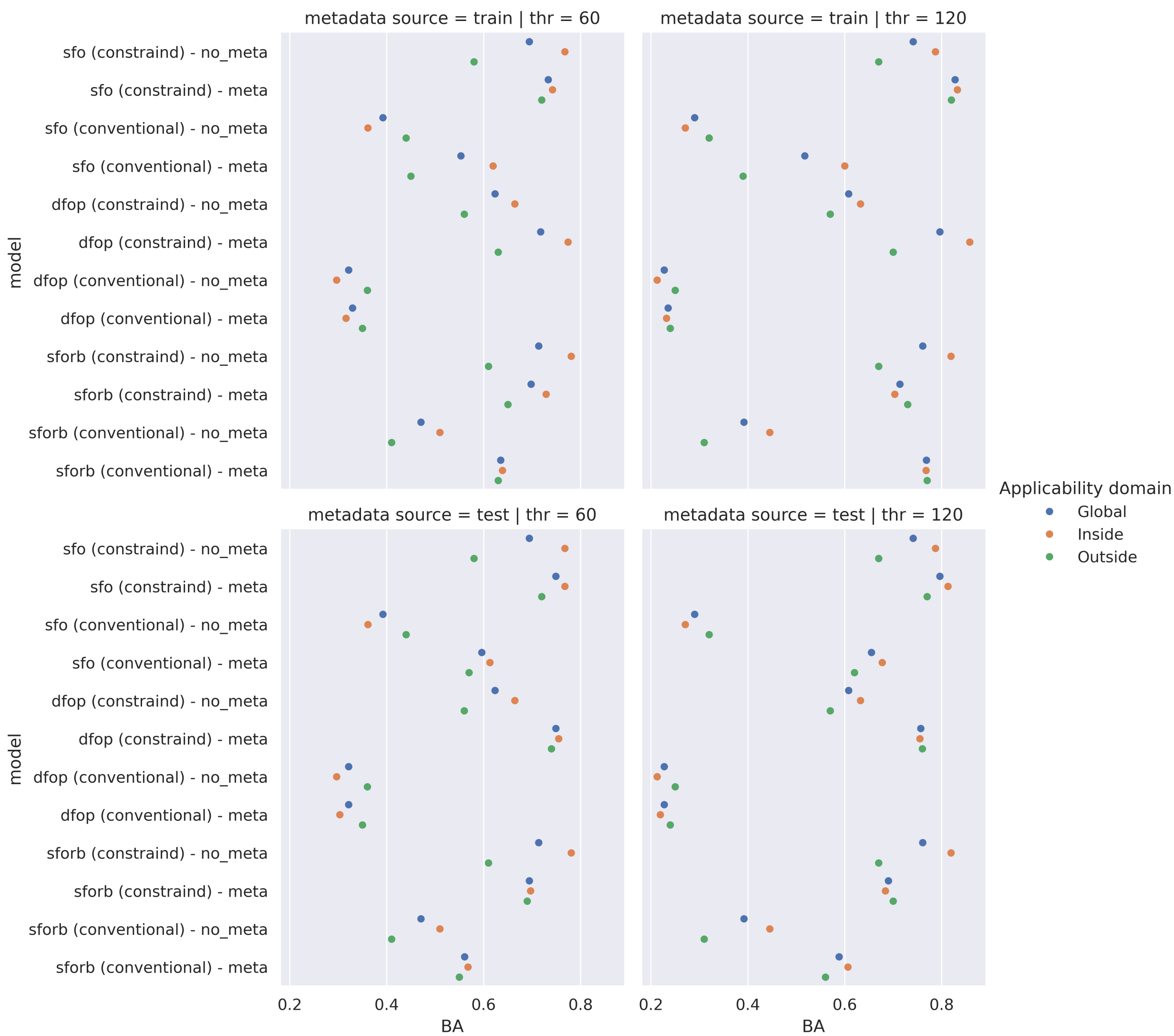

Figure S3: DT_{50} Classification performance on Time-Split Test Set (Balanced Accuracy, BA), comparing the best performing models (based on the MSE for the random Test Set), with and without access to soil metadata. Here, the results were independent of c0. Likewise, the use of Training Set imputed soil metadata vs Time-Split Test Set soil sample experimental metadata is denoted using metadata source = train vs test, respectively.

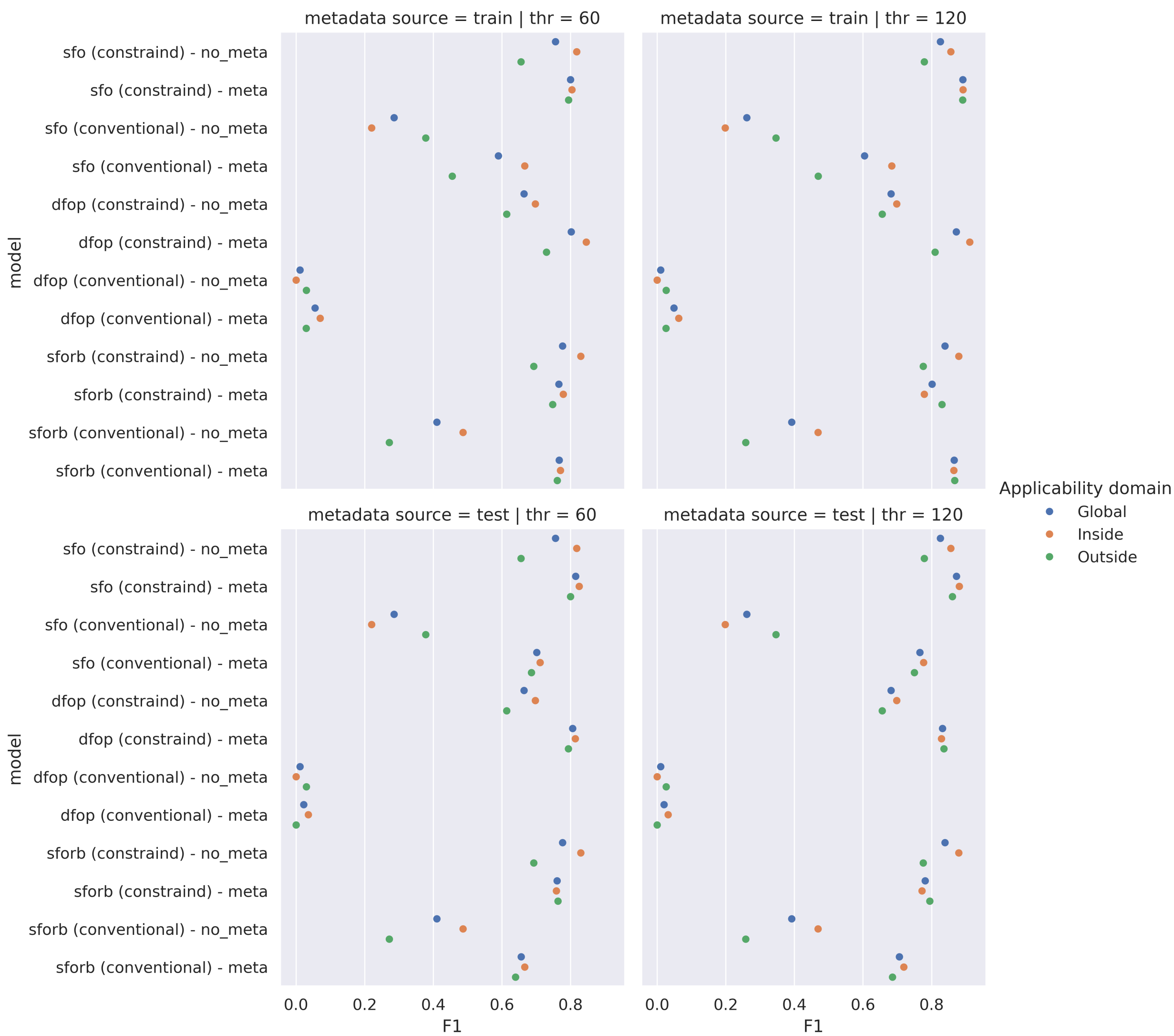

Figure S4: DT_{50} Classification performance on Time-Split Test Set (F1-Score, F1), comparing the best performing models (based on the MSE for the Test Set), with and without access to soil metadata. Here, the results were independent of c0. Likewise, the use of Training Set imputed soil metadata vs Time-Split Test Set soil sample experimental metadata is denoted using metadata source = train vs test, respectively.

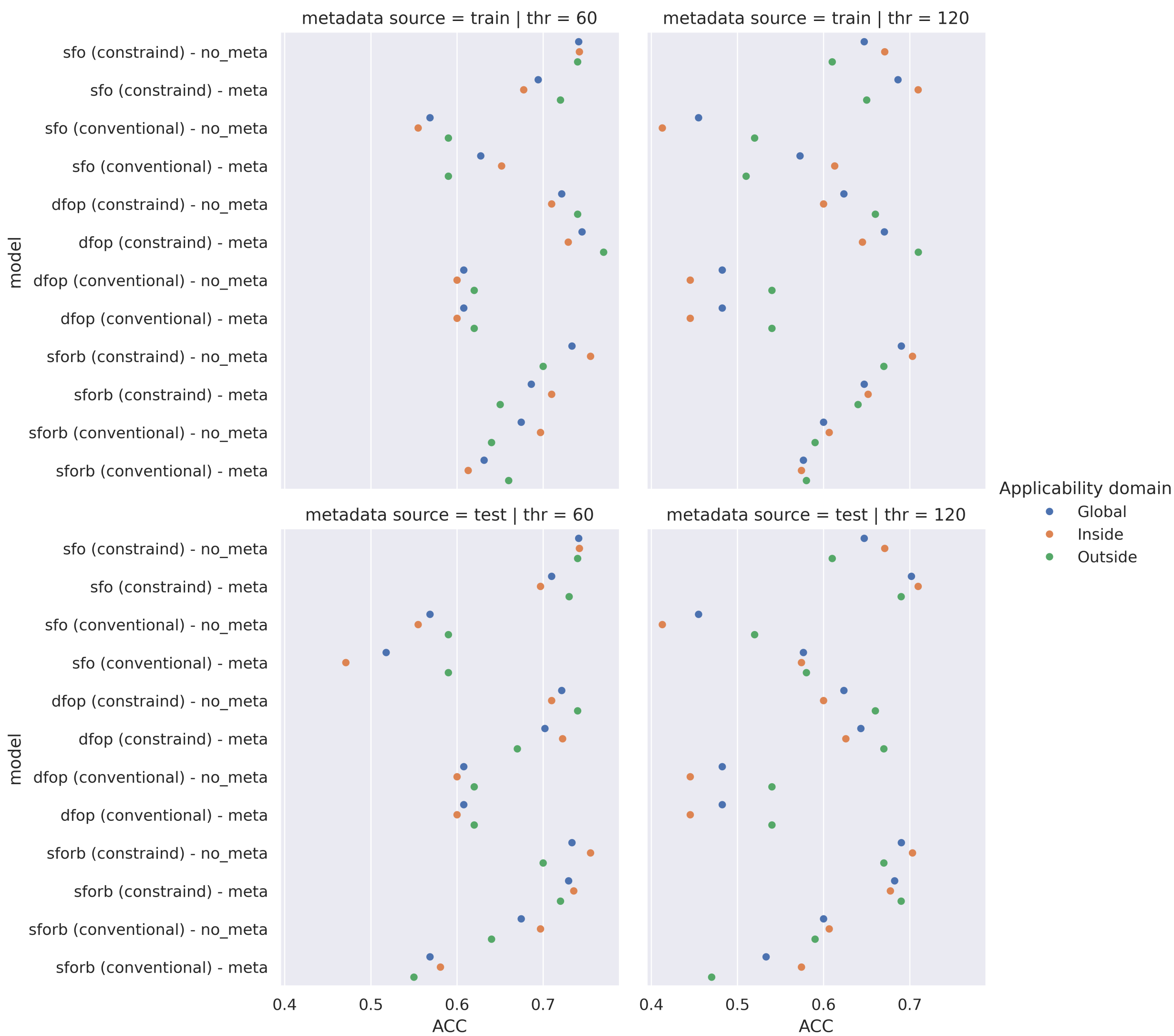

Figure S5: DT_{90} Classification performance on Time-Split Test Set (Accuracy, ACC), comparing the best performing models (based on the MSE for the Test Set), with and without access to soil metadata. Here, the results were independent of c0. Likewise, the use of Training Set imputed soil metadata vs Time-Split Test Set soil sample experimental metadata is denoted using metadata source = train vs test, respectively.

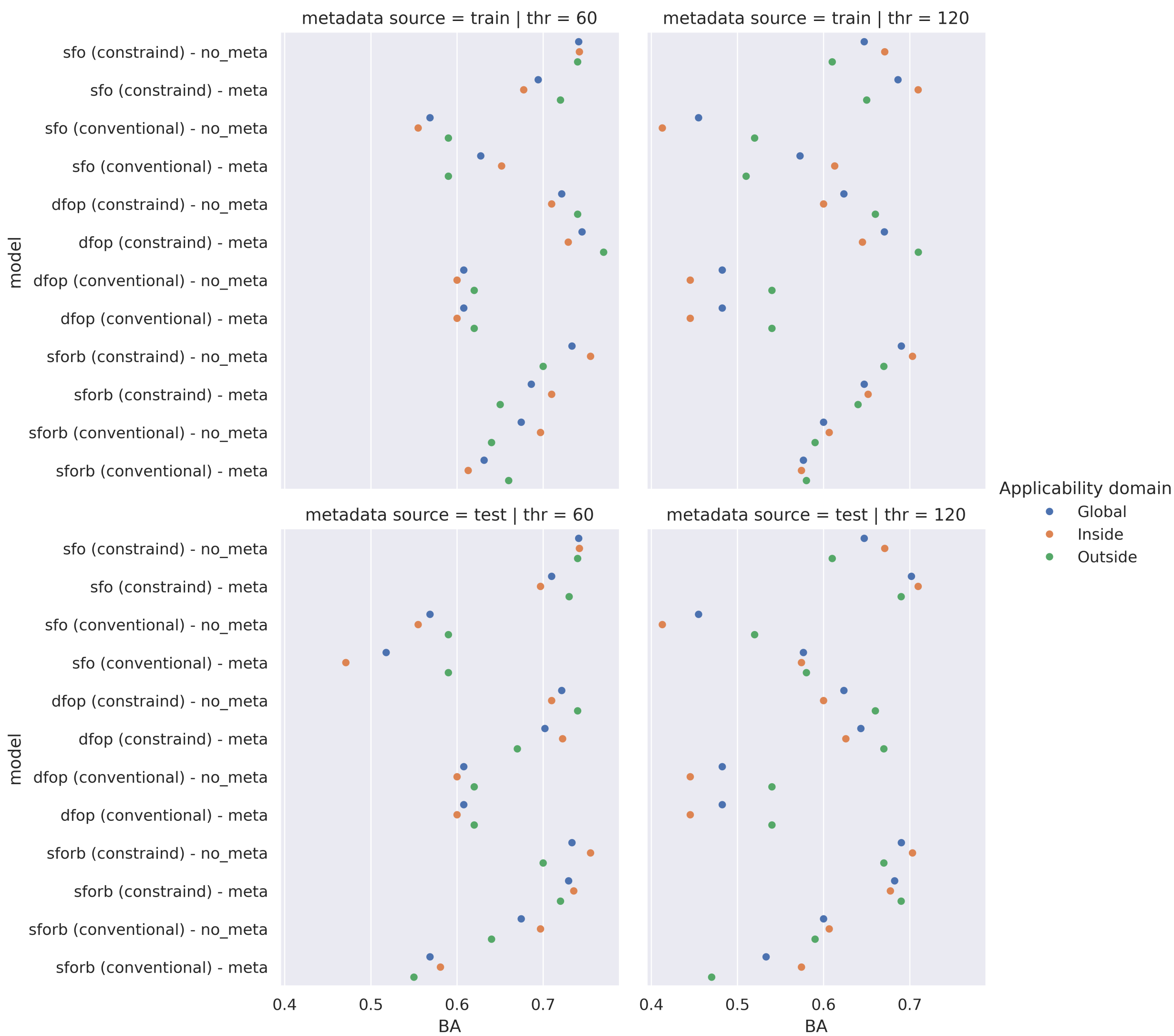

Figure S6: DT_{90} Classification performance on Time-Split Test Set (Balanced Accuracy, BA), comparing the best performing models (based on the MSE for the Test Set), with and without access to soil metadata. Here, the results were independent of c0. Likewise, the use of Training Set imputed soil metadata vs Time-Split Test Set soil sample experimental metadata is denoted using metadata source = train vs test, respectively.

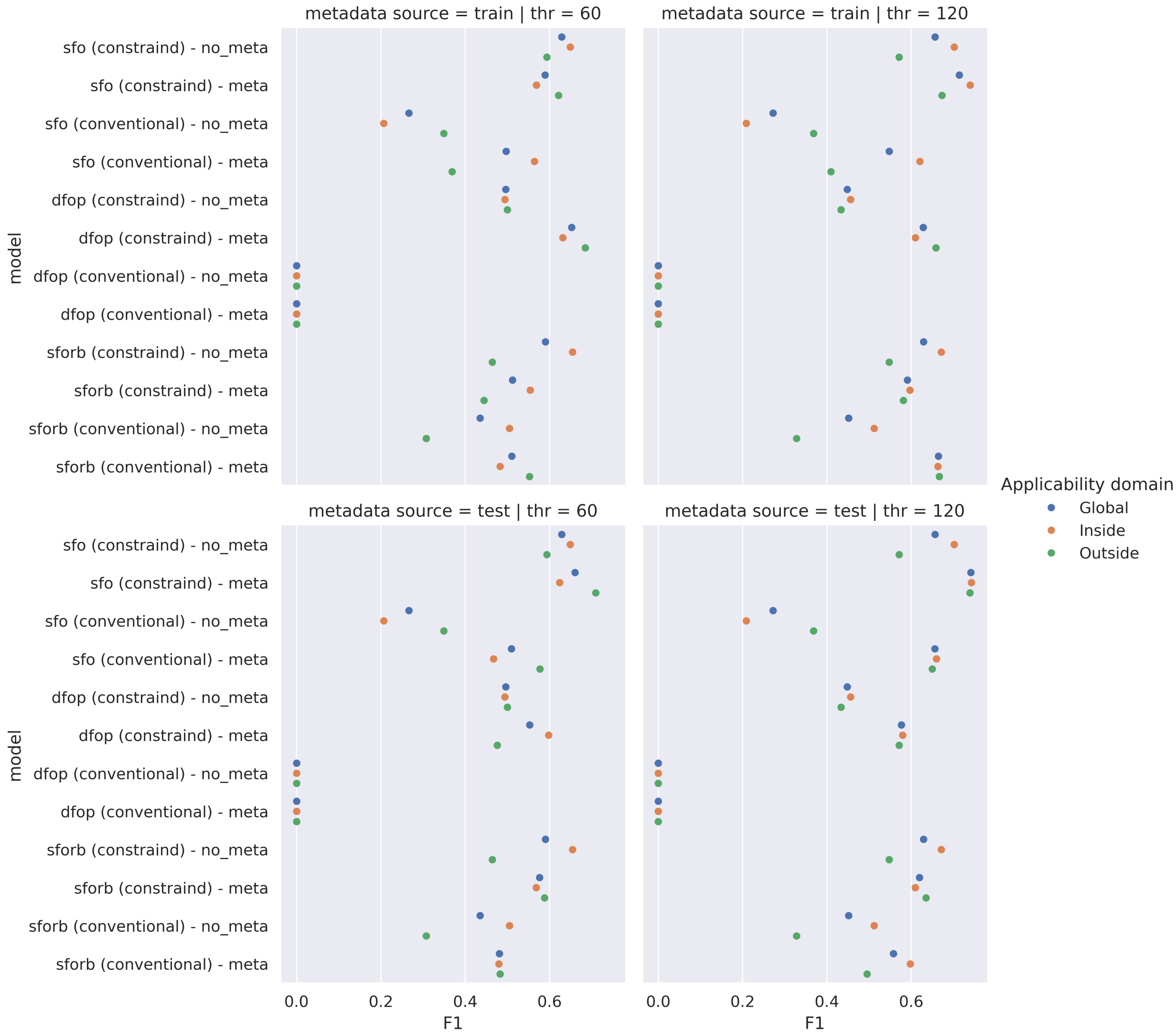

Figure S7: DT_{90} Classification performance on Time-Split test set (F1-Score, F1), comparing the best performing models (based on the MSE for the Test Set), with and without access to soil metadata. Here, the results were independent of c0. Likewise, the use of Training Set imputed soil metadata vs Time-Split Test Set soil sample experimental metadata is denoted using metadata source = train vs test, respectively.

References

(1) FOCUS Work Group on Degradation Kinetics. Generic Guidance for Estimating Persistence and Degradation Kinetics from Environmental Fate Studies on Pesticides in EU Registration. Version 1.1, 2014. https://esdac.jrc.ec.europa.eu/public_path/projects_data/focus/dk/docs/FOCUSkineticsvc1.1Dec2014.pdf (accessed 2026-03-06).

(2) Akiba, T.; Sano, S.; Yanase, T.; Ohta, T.; Koyama, M. Optuna: A Next-Generation Hyperparameter Optimization Framework. In *Proceedings of the 25th ACM SIGKDD International Conference on Knowledge Discovery & Data Mining*; KDD '19; Association for Computing Machinery: New York, NY, USA, 2019; pp 2623–2631. https://doi.org/10.1145/3292500.3330701.
